# Diploid dual assemblies reveal the telocentric structure and extensive allelic heterogeneity of canine genomes

**DOI:** 10.1093/nargab/lqag035

**Published:** 2026-04-23

**Authors:** Jeffrey M Kidd, Yassine Souilmi, Benjamin D Rosen, Ruqayya Khan, David Weisz, Olga Dudchenko, Erez Lieberman Aiden, Robert Zammit, J William O Ballard

**Affiliations:** Department of Human Genetics and Gilbert S. Omenn Department of Computational Medicine and Bioinformatics, University of Michigan, Ann Arbor, MI 48109, United States; Australian Centre for Ancient DNA, The University of Adelaide, Adelaide, South Australia 5005, Australia; Animal Genomics and Improvement Laboratory, Agricultural Research Service USDA, Beltsville, MD 20705, United States; The Center for Genome Architecture, Department of Molecular and Human Genetics, Baylor College of Medicine, Houston, TX, 77030, United States; The Center for Theoretical Biological Physics, Rice University, Houston, TX 77005, United States; The Center for Genome Architecture, Department of Molecular and Human Genetics, Baylor College of Medicine, Houston, TX, 77030, United States; The Center for Theoretical Biological Physics, Rice University, Houston, TX 77005, United States; The Center for Genome Architecture, Department of Molecular and Human Genetics, Baylor College of Medicine, Houston, TX, 77030, United States; The Center for Theoretical Biological Physics, Rice University, Houston, TX 77005, United States; The Center for Genome Architecture, Department of Molecular and Human Genetics, Baylor College of Medicine, Houston, TX, 77030, United States; The Center for Theoretical Biological Physics, Rice University, Houston, TX 77005, United States; Vineyard Veterinary Hospital, Vineyard, NSW 2765, Australia; School of Biosciences, University of Melbourne, Royal Parade, Parkville, Victoria 3052, Australia

## Abstract

Although an increasing number of long-read genome assemblies have been created from a diverse collection of dogs and wolves, most published assemblies represent the diploid genome as a single primary sequence. Here, we generate and analyze phase-resolved diploid dual assemblies from five canines. The most contiguous assemblies represent over half of the canine chromosomes as single contigs, permitting an assessment of the sequence and structure of canine chromosomes. Consistent with a telocentric classification, we find that the centromeres of canine autosomes begin an average of 59 kb from the start of the chromosome and are flanked by a 35 kb subtelomeric segment that is repeat-rich and shared across autosomes. Analysis of a pangenome graph constructed from the 10 haplotype-resolved assemblies shows that short tandem repeat loci are three times more common than variable number tandem repeat loci and that the landscape of canine structural variation features extensive allelic heterogeneity. The pangenome graph includes examples of complex, nested allelic variation involving SINEC (a carnivore-specific SINE) and LINE-1 mobile elements. Analysis of 3′ transductions implicate an uncharacterized source element with high activity and demonstrates the presence of full-length LINE-1s capable of retrotransposition that are segregating among canines.

## Introduction

The advent of long-read sequencing represents a revolution in genomics, enabling the near-routine construction and analysis of high-quality genome assemblies [1, 2]. However, the correct assembly of genomes composed of abundant duplicated and repeated sequences remains a challenge [3]. These challenges are exacerbated by the diploid nature of most sequenced samples, which require differentiation between paralogous and allelic variation [4]. Until recently, mammalian genome assemblies usually represented this complexity by generating a largely contiguous primary assembly that randomly switches between haplotypes and a second, highly fragmented alternative assembly designed to capture allelic variation [3, 5]. However, the paradigm of primary and alternative assemblies gives an incomplete representation of the genetic variation present in a diploid sample. True haplotype-resolved diploid assemblies can be generated through the analysis of trios or by coupling accurate long-read sequencing with information on chromosome phase, as determined by Hi-C or strand-seq approaches [4–8]. When accurate Hi-C or related data is unavailable, a dual assembly representing the two haplotypes of a diploid sample can still be generated using PacBio HiFi sequencing data [5]. Although the resulting dual assemblies may have imperfect phasing, they are often highly contiguous, accurate, and informative for downstream analyses of genomic variation [5].

The striking phenotypic variation found among modern dogs makes canines an attractive system for studying selection and the molecular basis of complex phenotypes [9–11]. Over the past five years, the canine research community has generated multiple genome assemblies using long-read sequencing technologies [12–22]. Pangenome graphs are a sophisticated framework for analyzing variation among genome assemblies [23]. Pangenome graphs provide an alternative to using a single linear reference genome, enabling sophisticated all-vs-all comparisons among samples. However, most of the existing canine genome assemblies consist of a primary assembly and represent diploid variation as highly fragmented collections of alternative contigs. This representation is not suitable for analysis using pangenome approaches. Despite this limitation, prior studies, which have relied mainly on direct pairwise alignments between genome assemblies, have clarified the extent of genomic variation among canines. A key finding of prior analyses is the striking contribution of dimorphic Long INterspersed Element-1 (LINE-1) and Short INterspersed Element (SINE) transposable elements to canine diversity [13, 24]. LINE-1s are autonomous retrotransposons that encode the proteins required for their mobilization [25]. In contrast, the retrotransposition of SINEs requires the proteins encoded by LINEs [26]. The dimorphic SINE in dog genomes is the carnivore-specific CAN-SINE or SINEC family, which arose from a tRNA-Lysine prior to the diversification of the order Carnivora into Feliformia (cat-like) and Caniformia (dog-like) species [27].

The canine karyotype consists of 38 pairs of autosomes along with the X and Y sex chromosomes. The autosomes feature a centromere located near the end of the chromosome in what has been described as an acrocentric or telocentric configuration [9, 28–30]. Telocentric and acrocentric are cytogenetic terms that describe the position of a centromere. In acrocentric chromosomes, the centromere is distinctly closer to one end of the chromosome, resulting in a long arm (the q-arm) and a short arm (the p-arm). In telocentric chromosomes, the centromere is adjacent to one end of the chromosome, yielding a p-arm that is not cytogenetically visible [31].

Centromeres, the regions of the chromosome where spindle microtubules associate, are typically composed of long segments of tandemly repeated sequence known as satellite repeats [32]. The key factor that determines centromere function is the presence of the histone H3 variant CENP-A [33]. Satellite repeats enriched at functional centromeres in dogs have been identified using CENP-A ChIP-seq [34]. Centromeric satellite repeat arrays often exhibit a complex pattern of organization and sequence identity that is evolutionarily dynamic [35, 36]. Prior studies have shown that a major component of canine satellite sequence is a ∼734 bp unit known as a Bsp repeat that is composed of divergent segments and contains an RNA polymerase III promoter, which generates abundant transcripts [37].

In this study, we describe the generation and analysis of diploid dual assemblies from five samples, including the generation of a new haplotype-resolved dual assembly using previously published data from a Greenland Wolf [19]. The best assemblies are highly contiguous, with more than half the chromosomes represented as single contigs. We assess the organization of the resulting assemblies, including 27 centromeres. We describe the telocentric configuration of autosomal centromeres and reveal extensive allelic heterogeneity among canine genomes using a pangenome graph.

## Materials and methods

### Animal ethics statement

No animals were housed as part of this study. Blood samples were collected from animals under protocols approved by the University of New South Wales.

### Data generation and genome assembly

PacBio High-Fidelity (HiFi) sequencing data were generated from a Siberian Husky (SH; individual name: Dite), an Australian Cattle Dog (ACD; individual name: Sonar), and a Chow Chow (CC; individual name: Magic) by DNA Link (South Korea). Illumina Hi-C sequencing data were generated for SH and ACD at the Australian Genome Research Facility (AGRF) using the Omni-C proximity ligation protocol (Dovetail Genomics). HiFi and *in situ* Hi-C Hi-C data were generated from a New Guinea Singing Dog (NGSD; individual name: Melody) from North Georgia Wildlife Park. For the NGSD, HiFi sequencing was performed at the University of Delaware, and Hi-C data were generated by the DNA Zoo Consortium (www.dnazoo.org). Hi-C data was not available for Magic, the Chow Chow. Haplotype-resolved dual assemblies were generated using HiFiasm [5] (version 0.25.0) with options –dual-scaf and –telo-m CCCTAA. Illumina Hi-C data were included for the ACD, SH, NGSD, and GW assemblies using the –h1 and –h2 flags. We note that a primary assembly of GW has been described [19] and that a primary assembly of NGSD was previously released through the DNA Zoo portal https://www.dnazoo.org/assemblies/canis_lupus_familiaris_ngsd.

### Scaffolding and assignment to chromosomes

For each haplotype-resolved dual assembly, sequences were assigned to chromosomes using ragtag version 2.1.0 [38]. We used the primary assembled chromosomes (chr1-chr38, chrX, and chrM) from the UU_Cfam_GSD_1.0 assembly [14], along with Y-chromosome sequences from a Labrador retriever, as a reference. This is the same reference used by the Dog10K Consortium [39]. This chromosome Y sequence is fragmentary and incomplete. One contig from the Siberian Husky (a female) had partial alignment to a Y chromosome contig; we reclassified the resulting contig as being unlocalized. The Greenland Wolf, a male sample, has a Y-chromosome sequence assigned to both the haplotype-1 and haplotype-2 assemblies. A full chrX sequence is included in the Greenland Wolf haplotype-1 assembly, while a 9 Mbp portion of the sequence is also assigned to chrX in the haplotype-2 assembly. Sequences not assigned to a chromosome and identified as likely contaminants by the NCBI contamination filter were removed [40].

### Mitochondria genome assembly

The mitochondrial genome was assembled from the raw HiFi reads for each sample using MitoHiFi (version 3.2.1) with the dog mitochondrial reference (accession NC_002008.4) used to gather reads for assembly [41, 42]. The resulting sample-specific mitochondrial sequences were searched against each genome assembly [43], and all contigs with 75% or more of their length encompassed by mitochondrial alignments were removed. The mitochondria assemblies are included in the haplotype-1 assembly for each sample.

### Assessing assembly quality and completeness

Assembly quality was assessed using Merqury (version 1ad7c32) and Meryl (version 1.4.1) with a k-mer size of k = 21 [44]. Since Illumina WGS data were unavailable, comparisons were based on k-mers present in the raw HiFi reads for each sample. The representation of single-copy genes was determined using compleasm [45] (version 0.2.7) with the carnivora_odb12 database [46].

### Annotation of repeated and duplicated sequences

Repetitive sequence was annotated using RepeatMasker (version 4.0.7) with the Dfam_Consensus-20170127 and RepBase-20170127 repeat databases and the option –species dog [47]. Tandem repeats were annotated using the trf program (version 4.04) with options ‘2 7 7 80 10 50 500′ [48]. Additional repetitive and low complexity sequence were annotated using the Windowmasker program (version 1.0.0) with option -dust true [49]. The density of telomeric repeats in 10 kb windows was determined using tidk (version 0.2.65) with options -s TTAGGG -w 10 000 [50].

Segmental duplications were identified in each assembly using the program BISER (version 1.4) with sequences identified by RepeatMasker or trf soft-masked [51]. Duplications with a length less than 1 kb or a divergence greater than 10% were removed. Duplications involving the mitochondria were also removed since nuclear mitochondrial insertions (Numts) arise via distinct mechanisms. Pairwise duplications were merged into non-redundant intervals using bedtools (version 2.30.0) [52]. To focus on the largest and most recent duplications, some analyses were limited to duplications with a length of 10 kb or greater and a divergence of 5% or less.

### Centromere analysis

Candidate centromere regions were defined using previously generated CENP-A ChIP-seq data (accession SRR499750) [34]. The ChIP-seq data were generated from a kidney cell line established from a Cocker Spaniel breed dog. Low-quality bases were trimmed from Illumina sequencing reads using Sickle (version 1.33, https://github.com/najoshi/sickle) with default parameters. The resulting reads were aligned to the CD.1 assembly using bwa mem [53] (version 0.7.17) with parameters -k 50 -c 1000000. Alignments were sorted and filtered to remove non-primary and supplementary alignments using samtools [54] (flag -F 2308). CENP-A ChIP-seq read depth was tabulated in 10 kb windows using mosdepth [55] (version 0.3.2). Candidate centromeres were identified as segments near the start of each chromosome with mean CENP-A ChIP-seq depth greater than 5 reads. Segments near each other were manually merged and further assessed based on the placement of assembly gaps and the presence of telomeric repeats in the proper orientation. We additionally annotated centromere structures based on the density of CarSat1, Carsat2, Bsp, and SAT1_CF satellite repeats using RepeatMasker [34] tabulated in 10 kb windows. Self-similarity plots of centromeres were generated using ModDotPlot [56] (version 0.9.4) with a window size of 1000. Sequence shared among assembled chromosomes was visualized using miropeats [57] with additional annotations based on RepeatMasker.

### Pangenome analysis

To estimate divergence levels, a 2Mb segment from the CanFam4/UU_Cfam_GSD_1.0 assembly (chr1:61 500 001–63 500 000), which does not overlap annotated segmental duplications [58], was aligned to each assembly using minimap2 (version 2.26) [59]. Two measures of identity were calculated from the resulting alignments: gap-excluded identity, which does not consider insertions and deletions, and gap-compressed identity, which considers consecutive insertions or deletions as a single difference.

Pangenome alignments were constructed using the Minigraph-Cactus pipeline (cactus version 2.9.8) [60]. Graphs were constructed separately for each chromosome, including sequence from the UU_Cfam_GSD_1.0 assembly as the reference sample, with the options: –vcf –giraffe –gfa and –gbz. Although UU_Cfam_GSD_1.0 was included in the resulting variant file, all analyses were limited to sites that were polymorphic among the haplotype-resolved assemblies (10 haplotypes for the autosomes and 9 for chrX). Visualizations were constructed using a sequence tube map [61].

We identified insertion and deletion structural variants by parsing the final variant file produced by the Minigraph-Cactus pipeline. We removed any locus that: ***(i)*** has a missing genotype in one of the analyzed haplotypes, ***(ii)*** is not variable among the five analyzed samples, or ***(iii)*** has a maximum difference in allele size among the analyzed samples of less than 50 bp. Next, we characterized the sequence content of identified structural variants based on methods previously used to analyze human and great ape pangenome graphs [62]. For each locus, we characterized the longest allele found among the five samples using RepeatMasker (version 4.0.7) with the Dfam_Consensus-20170127 and RepBase-20170127 repeat databases and the option –species dog [47]. We applied the symmetric DUST algorithm as previously implemented to further annotate low complexity loci [63]. Pure tandem repeats with a motif occurring two or more times were identified using the etrf program [62]. We joined together fragmented RepeatMasker annotations. Since SINEC elements feature an internal (CT)_N_ segment of variable length, segments inside of a SINEC that RepeatMasker annotated as (CT)n, (AG)n, (TC)n, or (GA)n were counted as part of the SINEC. Loci with a merged RepeatMasker annotation spanning at least 70% of the allele length were classified based on the annotated repeat type. Loci with a DUST, Simple_repeat, or Low_complexity annotation of at least 70% of the total allele length were classified as Low Complexity. Low Complexity loci were classified as VNTR if 70% of the allele length was encompassed by a tandem repeat annotation with a unit length of 7 bp or longer, and were classified as STR if 70% of the allele length was encompassed by a tandem repeat annotation with a unit length of 6 bp or shorter. Remaining loci with a union of DUST and RepeatMasker annotations spanning at least of 70% of the allele length were classified as Mixed, loci with annotations spanning at least 5% of the allele length were classified as Partial, and remaining loci were unclassified. SINEC, LINE/L1, and LTR/ERV annotations are shown separately, while other elements were combined into a single category.

### Enrichment of exact short tandem repeats relative to other annotations

The positions of exact tandem repeats with a repeat unit 6 bp or shorter were determined in the ACD.1 assembly and the human T2T assembly (GCA_009914755.4) using etrf. The overlap of the identified segments was determined relative to RepeatMasker annotations of each assembly. Enrichment relative to random expectations was determined using bedtools shuffle with 100 permutations. Since RepeatMasker element annotations may be interrupted by internal simple repeat annotations, adjacent element annotations that have the same element ID and are separated by a Simple_repeat segment were merged together.

### Additional SINEC and LINE-1 analyses

We performed additional analyses of loci annotated as SINEC or LINE-1 sequences. Analysis was limited to loci where all observed alleles are assigned to a SINEC or LINE-1 or to an empty site of exactly 1 bp in the reported VCF file. For SINEC loci, we tabulated the number of times that multiple alleles were observed at each locus as a measure of allelic heterogeneity. We additionally classified each allele as representing the ancestral (i.e. empty site) or derived (i.e. SINEC) state and examined profiles of allele sharing across autosomal loci. We searched for open reading frames in LINE-1 sequences at least 4 kb in length using the NCBI ORFfinder program version 0.4.3 [64] and compared predicted proteins with the ORF1p and ORF2p proteins from the L1-Y_Cf consensus [65] using BLAT [66]. We searched for LINE-1 alleles that contain 3′ transductions by extracting the sequence downstream of the longest LINE-1 annotation identified by RepeatMasker in the longest allele identified in the pangenome for each locus. Each candidate transduction was aligned against the UU_Cfam_GSD_1.0 assembly using blat [66] and annotated with both RepeatMasker and SDUST. We removed alignments to unplaced assembled chromosomes, alignments with a match score less than 25, alignments that do not include at least 25 unmasked positions in the query (based on the union of RepeatMasker and SDUST), and those aligned within 7 kb of the locus position. Transductions with an excess of alignments due to segmental duplication or homology to interspersed repeats, or with a fragmented alignment with a length in the genome that is 100 bp longer than the aligned query sequence, were also removed.

We estimated the rate of SINEC and LINE-1 insertion using a phylogenetic approach based on comparison between each assembly and GW.1. Analysis was limited to the autosomes, and regions annotated as being duplicated in CanFam4/UU_Cfam_GSD_1.0 were removed. This resulted in a genome size of 2 127 440 313 bp retained for analysis. For each comparison, we determined the number of single nucleotide differences between the assemblies and calculated the average time of genome divergence in generations, assuming a mutation rate of 4.5*10^−9^/bp/generation [67]. Next, we determined the number of mobile element insertions that occurred in each genome relative to GW.1 and combined the two values to obtain an estimate of the rate of insertions per generation. For LINE-1s, we removed variants with a RepeatMasker annotation that ended more than 100 bp from the 3′ end of the element consensus. This filter suppresses *intra*-LINE-1 deletion variants that likely did not form as new insertions. To avoid double-counting insertions that occurred in GW.1, rate estimates were tabulated as the mean among 10 comparisons: elements present in each of the 9 assemblies and absent in GW.1, along with elements in GW.1 and absent from the ACD.1 assembly.

## Results

### Generation of highly complete haplotype-resolved dual assemblies from five canines

By combining the latest algorithms for genome assembly with high-quality PacBio HiFi-long read sequencing data and Illumina Hi-C chromosome conformation data, we generated phase-resolved dual genome assemblies from five canines. This includes new diploid assemblies from four dogs (Australian Cattle Dog [ACD], Chow Chow [CC], Siberian Husky [SH], and New Guinea Singing Dog [NGSD]) as well as a new dual assembly generated from previously published data for a Greenland Wolf [19] (GW, Table 1). Analysis of the representation of k-mers in each assembly and in the corresponding raw HiFi reads, along with analysis of single-copy gene orthologs, shows that the 10 assemblies are of high quality and completeness (Table 1, Supplementary Fig. S1). As expected, the GW.1 and GW.2 assemblies are each slightly more fragmented than the published pseudo-haploid assembly of this sample (Table 1 and Supplementary Table S1). The ACD and NGSD assemblies have gene completeness metrics comparable to those of the UU_Cfam_GSD_1.0 assembly, which served as the reference for a recent large-scale analysis of canine genome variation, and the Clu-1 assembly, which was generated from a coyote-wolf hybrid [14, 21, 39]. The ACD.1 and ACD.2 assemblies, which were generated using the greatest read depth, show exceptional continuity with over half the chromosomes present as single, gap-free contigs.

**Table 1.** Summary of five haplotype-resolved canine assemblies

	Australian Cattle Dog		Chow Chow		Siberian Husky		New Guinea Singing Dog		Greenland Wolf	
	ACD		CC		SH		NGSD		GW	
HiFi N_50_	17 980		18 835		17 097		14 659		13 402	
HiFi coverage	123.2		24.4		18.8		28.9		34.6	
	**ACD.1**	**ACD.2**	**CC.1**	**CC.2**	**SH.1**	**SH.2**	**NGSD.1**	**NGSD.2**	**GW.1**	**GW.2**
*k*-mer completeness (%)	97.44	97.42	96.32	96.11	97.17	97.63	98.99	98.9	97.01	92.34
Single copy gene (%)	98.75	98.70	96.76	96.92	97.20	97.71	98.70	98.73	98.46	95.05
QV	70.5	70.7	65.6	66.0	67.2	67.0	67.4	67.5	67.6	67.8
Gap-free chromosomes	21	20	0	0	0	0	9	7	2	1

Statistics are provided for PacBio HiFi reads and the resulting assemblies obtained from five canines as indicated. GW data was reprocessed from Sinding *et al*. N_50_: the length of the shortest read such that 50% of the total data is made up of reads that length or longer. Estimated total coverage was obtained by dividing the total HiFi read length by an estimated genome size of 2 350 000 000 bp. *k*-mer completeness and quality values (QV) were obtained by comparing each assembly with the PacBio HiFi reads used to generate it using Merqury. The fraction of intact single-copy genes represented in each assembly was obtained using compleasm with the carnivora_odb12 database of protein-coding genes. The GW.2 assembly does not include the X chromosome.

### Landscape of chromosomal features in a highly contiguous Australian Cattle Dog assembly

Due to its high contiguity, we focused our analysis on the ACD.1 assembly, which includes 21 chromosomes represented as single contigs. First, we identified segmental duplications in the ACD.1 assembly based on genome self-alignment, finding that 5.9% of the assembled chromosome sequence is included in an identified duplication (Supplementary Table S2). To provide a view of recent, large segmental duplications, we next considered the 13 434 duplication pairs that involve only assembled chromosomes and are at least 10 kb in size, with a maximum divergence of 5%. This set encompasses 4.6% of the assembled chromosome sequence. Of the duplication pairs, 7.6% (1 022 of 13 434) are intrachromosomal duplications with 40.5% (414 of 1 022) of intrachromosomal duplications having a tandem configuration with a separation of less than 200 000 bp between paralogs, while the remaining 59.5% (608 of 1 022) of the pairs are dispersed (Supplementary Fig. S2). Interchromosomal duplication pairs are concentrated at the centromeric end of the autosomes, with 87% of interchromosomal duplication pairs (10 788 of all 12 412 interchromosomal pairs) involving paralogs that are both located within the first 5 Mbp of an autosome.

**Figure 1.**
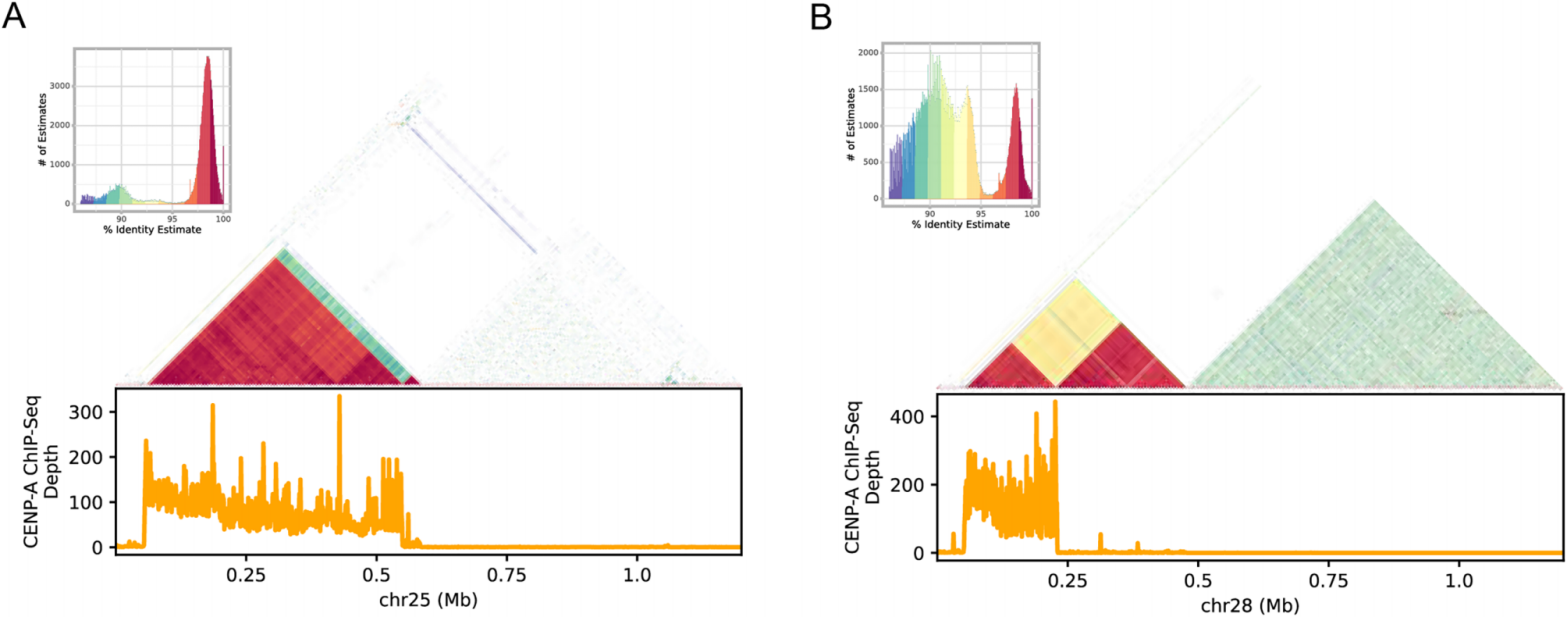
Structure of two canine centromeres. Self-similarity plots of the first 1.2 Mb of chr25 (**A**) and chr28 (**B**) from the ACD.1 assembly are depicted. Plots were generated using ModDotPlot with a *k*-mer size of 21 and a window size of 1000. For each column, the inserted histogram shows the distribution of *k*-mer identities. The triangle plot illustrates the self-similarity of each region, with *k*-mers coloured according to the histograms. The distribution of CENP-A ChIP-seq coverage in 10 kb windows is plotted below each triangle plot. The chr25 centromere region features a single large array of satellite sequence with elevated CENP-A ChIP-seq coverage. The chr28 centromere features a more complex structure that includes three arrays of satellite sequence with distinct sequence identity. The CENP-A ChIP-seq signal is found only at the first array.

To define the location of centromeres, we mapped existing canine CENP-A ChIP-seq data to the ACD.1 assembly [34]. Candidate centromeres were identified as regions of elevated ChIP-seq signal. By comparing CENP-A ChIP-seq read depth with the location of assembly gaps, satellite sequences, and telomere repeats, we identified 27 centromeres that appear to be correctly assembled (Supplementary Table S3). This includes centromeres from 26 autosomes that begin an average of 59 kb from the start of the chromosome. Self-similarity analysis of the centromere sequence reveals a complex structure of tandem repeats of varying identity corresponding to the CENP-A ChiP-seq signal that is concentrated at the beginning of the chromosome (Fig. 1, Supplementary Fig. S3). For example, the chr25 centromere region features a single large array of satellite sequence with elevated CENP-A ChIP-seq coverage. The chr38 centromere features a more complex structure that includes three arrays of satellite sequence with distinct sequence identity. The CENP-A ChiP-seq signal is found only at the first array. The telocentric regions consist of telomeric repeats and a ∼35 kb subtelomeric segment that is rich in interspersed repeats and is shared among assembled chromosomes, followed by satellite sequence and simple repeats extending into the centromere (Supplementary Fig. S4). Although gaps are often coincident with centromeric satellites across all assemblies, some candidate centromeres appear to be contiguous in multiple samples (Supplementary Fig. S5). Comparison of chr25 between ACD.1 and GW.1 reveals a broadly similar centromere structure with an expanded repeat array present in GW.1 (Supplementary Fig. S6). Additionally, the GW.1 centromere contains an insertion of a LINE-1 element that is absent in ACD.1.

### Variants identified using a pangenome graph

**Figure 2.**
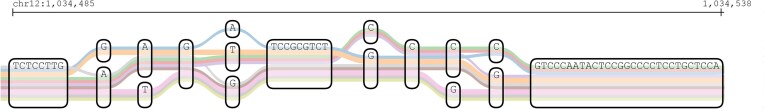
Variation among assembled genomes revealed by a pangenome graph. A sequence tube map view of the pangenome graph corresponding to a portion of exon two of the *DLA-88* gene is shown (UU_Cfam_GSD_1.0 chr12:1 034 484–1 034 538). The 10 lines correspond to the UU_Cfam_GSD_1.0 reference, along with 9 haplotypes assembled from the five analyzed individuals (this region is missing in the CC.1 assembly). There are five distinct haplotypes present among the 9 genomes assembled for this region. None of the assembled haplotypes matches the UU_Cfam_GSD_1.0 reference (silver line) across this region.

To take advantage of the power of phase-resolved genome assemblies for the analysis of structural variants, we created a pangenome alignment of the five diploid assemblies using Minigraph-Cactus [60]. We included the UU_Cfam_GSD_1.0 assembly as the reference in the pangenome graph, allowing direct analysis of haplotype variation at particular loci (Fig. 2).

Sequence alignment indicates that each diploid assembly has a sequence identity of over 99.75% relative to UU_Cfam_GSD_1.0 (Supplementary Table S4), supporting the use of the Minigraph-Cactus pipeline, which was designed for within-species pangenome graph construction. To account for differences in assembly quality and to limit the reliance on the UU_Cfam_GSD_1.0 reference, we limited subsequent analysis to loci that are variable among the five analyzed genomes and where there is no missing data. This graph includes 9 632 512 single nucleotide differences (SNPs) among the five assembled genomes that meet these criteria.

**Figure 3.**
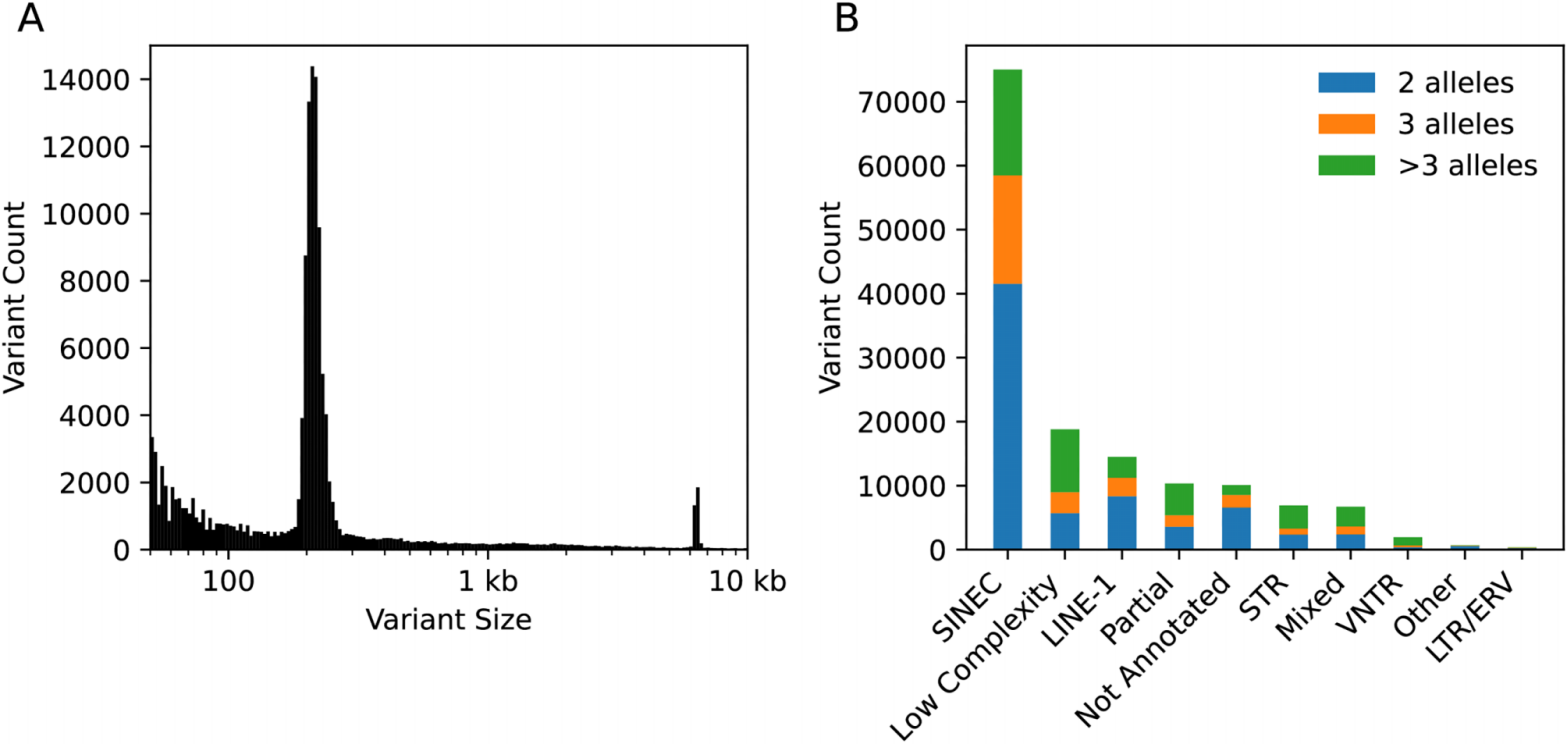
Spectrum of insertion and deletion structural variants identified using a pangenome graph. (**A**) A histogram of the size difference between the largest and smallest allele identified at 145 294 structural variants is shown. This histogram is plotted using bins of equal size on a log scale. (**B**) Classification of identified structural variants. The count of variants classified into 10 categories based on the sequence of the longest allele at each locus is shown. The number of sites that contain only two alleles is shown in blue, sites with three alleles in orange, and sites with more than three alleles in green. Low Complexity loci were classified as VNTR if 70% of the allele length was encompassed by a tandem repeat annotation with a unit length of 7 bp or longer, and were classified as STR if 70% of the allele length was encompassed by a tandem repeat annotation with a unit length of 6 bp or shorter.

We additionally identified 145 294 insertion and deletion structural variants in the pangenome graph where the alleles observed among the five samples differ by 50 bp or more in length. Consistent with the known contribution of LINE-1 and SINEC sequences to canine genomic diversity [13, 24], the structural variation size spectrum has distinct peaks at ∼200 bp and ∼6 kbp (Fig. 3A, Supplementary Fig. S7). We grouped the 145 294 structural variants into ten categories based on the sequence composition of the longest allele (Fig. 3B). Using a 70% composition criterion, 52% of loci were classified as SINEC variants (75 006 loci), followed by variants consisting of low complexity sequence (∼13%; 18 797 loci) and LINE-1 variants (∼10%; 14 486 loci). In contrast to the profiles found in human and great ape pangenomes [62], STR loci are more than three times as common as VNTR loci (6899 loci versus 1929 loci, respectively) among the analyzed canine genomes. STR loci are distributed across all canine chromosomes without a consistent enrichment toward chromosome ends (Supplementary Fig. S8). To further explore this pattern, we identified all short tandem repeats in the ACD.1 genome, defined as exact tandem repeats with a repeat unit of 6 bp or shorter, and compared them to other genome annotations. This revealed that canine short tandem repeat sequences are located within SINEC elements 4.6 times more frequently than expected by chance. We additionally classified all loci based on the number of distinct alleles represented in the pangenome graph, finding that nearly half of the loci are multiallelic (71 682 loci) even though only five individuals were analyzed.

### SINEC allelic heterogeneity and allele sharing

Given their disproportionate contribution to canine genome diversity, we performed additional analyses of SINEC variants. We identified 72 004 loci where each allele corresponds to either the SINEC sequence or to a pre-insertion empty site. One third of these loci are singletons, where only a single insertion allele is observed among the five analyzed genomes. Multiple alleles were present for 67% of the 47 977 non-singleton SINEs. Dimorphic SINEs are valuable markers for phylogenetic and population genetic analysis since the ancestral state of each locus is known to be the empty or non-insertion allele, and the presence of the element in an individual represents identity by descent [68]. We therefore analyzed the pattern of allele sharing among 69 384 autosomal SINEC loci. We limited analysis to the autosomes because the X chromosome has a ploidy of one in males and evolves with a different effective population size. The New Guinea Singing Dog contains a notable deficit of heterozygous insertions and an excess of homozygous insertions, consistent with the high degree of inbreeding in the captive NGSD population [69] (Supplementary Fig. S9). Nearly half (33 383) of the SINEC insertions are found in only a single individual. As expected from the history of dogs and wolves, 37% of the SINECs found in GW are exclusive to that sample. Approximately 42% of SINECs present in GW are also found in CC, CD, and SH, while only 37% are shared with NGSD, likely reflecting the reduced diversity in NGSDs (Supplementary Fig. S9).

### LINE-1 allelic complexity resolved by pangenome graphs

**Figure 4.**
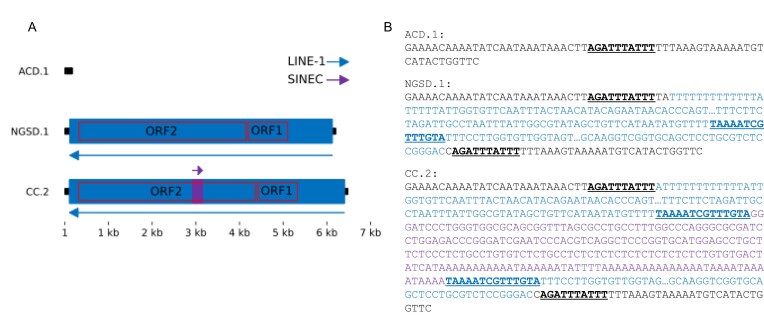
Nested structure of a dimorphic LINE-1 and SINEC that retain open reading frames. Locus chr1:380 121 contains three structurally distinct alleles. A schematic of the structure is depicted in (**A**). The empty site is represented by a sequence from the ACD.1 assembly. The NGSD.1 assembly contains a full-length LINE-1 insertion with two intact open reading frames. The CC.2 assembly contains the full-length LINE-1 that also has a SINEC element inserted into the ORF2 region. The LINE-1 and SINEC sequences are in opposite orientations. An annotation of the features of these alleles is shown in (**B**). The target site duplication corresponding to the LINE-1 insertion is shown in underlined black text and is present once in the ACD.1 allele and two times in the NGSD.1 and CC.2 alleles. LINE-1 sequence in the NGSD.1 and CC.2 alleles is colored blue. The CC.2 sequence contains a SINEC element inserted into the locus. SINEC sequence is colored in purple, and the target site duplication associated with the SINEC is depicted in blue underlined text. This sequence is present once in the NGSD.1 allele and twice in the CC.2 allele.

Full-length LINE-1s encode two proteins, ORF1p and ORF2p, required for their own mobilization. We identified 2 366 dimorphic loci with a LINE-1 allele that has intact open reading frames for both ORF1p and ORF2p. This includes a locus that has a remarkable nested configuration that contains three structurally distinct alleles: the empty site allele, a full-length LINE-1 insertion with intact open reading frames, and a full-length LINE-1 that additionally contains a SINEC insertion into ORF2p that retains an open reading frame (Fig. 4). Examination of the raw HiFi reads confirms the existence of all three alleles of this Matryoshka or nested structure (Supplementary Fig. S10). The SINEC insertion results in the addition of 78 amino acids into the predicted ORF2p sequence. The SINEC insertion occurred between the endonuclease and reverse transcription domains; in the human ORF2p, this region contacts the RNA template and has been labeled the tower domain [70], making it unlikely that the expanded ORF2p sequence retains function.

### Segregating LINE-1s that are capable of retrotransposition

**Figure 5.**
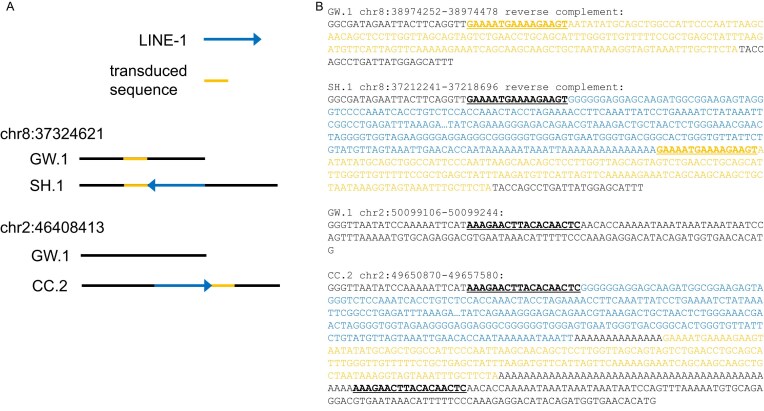
Parentless transductions demonstrate the presence of segregating LINE-1s capable of retrotransposition in canines. A schematic of a resolved parentless transduction is shown in (**A**). The CC.2 assembly contains a LINE-1 at chr2:46 408 413 (blue arrow). This insertion includes a 3′ transduction that maps to chr8 (orange segment). No LINE-1 is found at the chr8 locus in either the GW.1 or UU_Cfam_GSD_1.0 assemblies, making this a “parentless” transduction. However, the SH.1 assembly contains a full-length LINE-1 with open reading frames at the chr8 locus. Sequence features of the relevant alleles are shown in (**B**). Sequence from chr8 involved in the transduction is shown in gold text, LINE-1 sequence is shown in blue text, and sequence making up target site duplications is shown in bold underlined text. In each case, GW.1 contains the non-insertion or empty-site allele.

LINE-1 transcripts often extend beyond the polyadenylation signal encoded within the element, resulting in the inclusion of flanking genomic sequence in the retrotransposed RNA that can be used to identify the progenitor loci of individual LINE-1 insertions [71–76]. We identified 322 LINE-1 loci with 3′ transductions that were aligned to the UU_Cfam_GSD_1.0 assembly (Supplementary Table S5). This set includes a group of 10 LINE-1s that share a common transduced sequence, suggesting the presence of an uncharacterized source element with high activity. We previously described the presence of “parentless” 3′ transductions, which indicated the presence of segregating LINE-1 sequences capable of retrotransposition that have yet to be characterized [13]. The inclusion of multiple genomes in a single analysis clarifies this phenomenon. For example, we identified a full-length LINE-1 insertion on chr2 that was found only in CC (Fig. 5). This insertion includes a 3′ transduction that maps to a locus on chr8 that is not adjacent to a LINE-1 in the UU_Cfam_GSD_1.0 assembly and would therefore be classified as a parentless transduction. However, the pangenome includes a dimorphic LINE-1 at this locus with intact open reading frames. Transduction analysis shows that this dimorphic element gave rise to the chr2 insertion, demonstrating the presence of retrotransposition-capable LINE-1s that are segregating among canines.

### SINEC and LINE-1 mobilization rates estimated using diploid assemblies

We previously estimated the rate of SINEC and LINE-1 mobilization in canines using a phylogenetic approach calibrated using an estimated SNP mutation rate of 4.5*10^−9^/bp/generation [67, 77]. This prior analysis was based on alignment of pseudo-haploid assemblies from multiple canines, and may be biased by the non-random inclusion of heterozygous SINEC or LINE-1 insertions in the generated assemblies. To assess this, we estimated mobile element insertion rates by comparing each phased assembly with the GW.1 assembly. To account for a class of variants that arise via *intra*-LINE-1 deletion [77], we only considered LINE-1 variants with a RepeatMasker annotation that ended within 100 bp of the 3′ end of the LINE-1 consensus. Using these data, we estimate that a new SINEC insertion occurs in ∼1/15 births and a new LINE-1 insertion occurs in ∼1/117 births (Supplementary Table S6). These values are slightly higher than the estimates of ∼1/18–1/22 births for SINECs and ∼1/130–1/184 for LINE-1s reported in Blacksmith *et al*. [77].

## Discussion

Advances in sequencing technology and assembly algorithms permit the construction of diploid genome assemblies without parental information [5]. In this study, we describe dual assemblies produced from five canines, including a dual assembly generated from previously published HiFi and Hi-C data from a Greenland Wolf. Our dataset includes a highly contiguous assembly generated from an Australian Cattle Dog with deep HiFi sequencing. Although more than half the chromosomes in the resulting dual assembly are represented in single contigs, we avoid the term T2T when describing these assemblies. Although the ACD assembly is of high-quality, T2T implies a perfectly contiguous, correct, and complete assembly. Close examination of our assemblies reveals multiple regions of apparent misassembly, particularly near some centromeres and the largest duplications. Refinement of the existing assemblies to true T2T status is likely to require specialized sequence polishing approaches and the generation of additional data using an ultra-long read sequencing methodology (i.e. >100 kb long reads) [3, 78]. Despite this limitation, the assemblies described in this manuscript enable a detailed assessment of previously unexplored aspects of canine genome structure and facilitate a systematic assessment of structural variation using pangenome graphs.

In the ACD.1 assembly, we found that 87% of interchromosomal duplications map within the first 5 Mbp of the autosomes, a region known to contain the centromere. Our analysis of existing CENP-A ChIP-seq data suggests that 26 autosomal centromeres are well represented in the ACD.1 assembly. These centromeres have a complex structure of repetitive satellite sequence and begin an average of 59 kb from the start of the chromosome. The short distance between the 5′ telomere and the centromere suggests that canine chromosomes may be best thought of as telocentric. The telocentric autosomes contain a subtelomeric segment between the 5′ telomere and the centromere composed of ∼35 kb of repeat-rich sequence that appears to be shared across canine autosomes. This shared sequence, along with other shared features of centromere satellite arrays, may provide the homology needed for the formation of Robertsonian translocations that have been described in dogs [79]. Both centromeric and subtelomeric sequences are known to have a dynamic pattern of evolution leading to sequence and structural differences within and among closely related species [80–82]. For example, a recent study constructed near-T2T assemblies of C57BL/6J and CAST/EiJ mice, which also have telocentric autosomes [83, 84]. This study found that C57BL/6J telocentric chromosome ends contain a defined sequence structure that is shared among most mouse autosomes. In contrast, CAST/EiJ telocentric ends are larger and contain an expanded satellite repeat that is not found in C57BL/6J. Future long-read analyses will be required to determine the extent of centromere and subtelomere conservation among canines and related carnivores. The results we describe are based on existing CENP-A ChIP-seq data that was generated from a Cocker Spaniel breed dog. We focused our analysis on ACD.1 because it is the assembly with the greatest contiguity. A comparison of chr25 shows that although there are some differences, including an expanded array and the presence of a LINE-1 sequence, the broad centromere structure at this locus is conserved between a breed dog and a wolf. Future studies that directly measure CENP-A occupancy and DNA methylation across a diverse collection of samples may reveal additional aspects of centromere evolution in carnivores.

Perhaps the most important advantage of adopting a dual assembly approach is the ability to generate comprehensive maps of genomic variation using a pangenome graph [5, 60]. The pangenome we constructed fully represents 145 294 insertion and deletion structural variants. Three observations stand out from this graph. First, consistent with prior genome-wide comparisons, dimorphic mobile element insertions are the dominant type of structural variation [13, 24, 85]. Second, the pangenome graph reveals a notable amount of allelic heterogeneity, particularly for mobile element insertions. This heterogeneity reflects mutations that have arisen in the element sequence following its insertion at a locus. Heterogeneity is most apparent among SINECs, which feature a variable (CT)_N_ microsatellite segment and an A-rich tail [86, 87]. Although the generated assemblies are generally of high sequence quality (Table 1 and Supplementary Fig. S1), uncorrected errors in these segments may contribute to the apparent SINEC heterogeneity [88]. Allelic heterogeneity is also present among dimorphic LINE-1 insertions, including changes that disrupt the open reading frames of some alleles. Some heterogeneity is more complex. Reflecting the high frequency of dimorphic SINECs in canines, we resolved a remarkable Matryoshka or nesting structure that features a dimorphic SINEC that inserted into a dimorphic LINE-1.

Finally, in contrast to pangenome graphs generated from human and non-human primates [62], the canine pangenome contains an excess of STR loci as compared to VNTR loci. The predominance of STR loci in canines has been previously noted, along with an increased representation of pure mono-, di-, tri-, and tetranucleotide repeats [89]. Exact short tandem repeats are found throughout the genome and show a 4.6-fold enrichment in SINEC elements. A similar analysis using the T2T human genome reference shows a four-fold enrichment of exact short tandem repeats in satellite sequences and a two-fold enrichment in *Alu* elements, a type of SINE that is active in primates. Thus, in addition to their direct effect on canine variation, SINEC elements have also contributed to the proliferation of exact short tandem repeats throughout the canine genome.

Using diploid assemblies, we estimate that new SINEC and LINE-1 insertions occur in ∼1/15 and ∼1/117 births, respectively. Several caveats apply to these estimates, including the reliance on a single point estimate of the canine SNP mutation (here, assumed to be 4.5*10^−9^/bp/generation) and the assumption of a constant rate across lineages and the absence of selection. Blacksmith *et al*. performed a detailed assessment of element features, including target site duplications, poly (A) tails, and the inferred endonuclease cleavage site consensus sequences and reported both conservative and relaxed rate estimates [77]. In this study, we utilized variant annotations obtained from RepeatMasker annotations of the pangenome graph and only performed additional filtering to remove a class of structural variants involving LINE-1s. This class represents true structural variants involving LINE-1 sequence that arose from a deletion of sequence within an existing element. We removed these variants by requiring that LINE-1s annotations end within 100 bp of the 3′ end of the element consensus sequence. This filter removed 19.2% of all LINE-1 variants identified in the pangenome. A clear excess of the filtered loci is located within a LINE-1 sequence annotated in the UU_Cfam_GSD_1.0 assembly (36.7% of loci in an existing LINE-1 were filtered compared to 7.1% of loci not in an existing LINE-1, Fisher’s exact test *P *< 2.2*10^−16^). Overall, the estimates are similar among phased assemblies, with average estimates slightly greater than the rates reported in Blacksmith *et al*. This may reflect the increased representation of mobile element variants in the high-quality assemblies described in this study. In addition, estimated rates, particularly for LINE-1, are sensitive to filtering criteria. The variant classifications described in Fig. 3 are merely a first step enabled by pangenomes. Additional analyses of mutational processes in canines will benefit from careful consideration of factors known to influence mutagenesis, such as features of mobile element biology and STR sequence composition.

Until recently, most vertebrate genome assemblies have offered a biased representation of the diploid nature of genomes. Dual assembly approaches that fully represent both haplotypes enable the most robust assessment of genome structure and variation [3, 5]. Although the benefits of pangenomes are clear, several challenges remain, including the identification of inversion polymorphisms [23, 90]. We anticipate that improved technologies will soon enable a truly comprehensive view of the structure, variation, and evolution of the canine genome.

## Supplementary Material

lqag035_Supplemental_Files

## Data Availability

PacBio HiFi data and Illumina Hi-C data are available from the NCBI sequence read archive (SRA) under BioProject PRJNA1285458. Genome assemblies are available from GenBank under the following accessions: JBPXEE000000000, JBPXEF000000000, JBPXEG000000000, JBPXEH000000000, JBPXEI000000000, JBPXEJ000000000, JBPXEK000000000, JBPXEL000000000, DBJLPC000000000, and DBJLPD000000000. A VCF file describing variation among the assembled genomes is available from the Zenodo data archive under doi: https://doi.org/10.5281/zenodo.15881717.
