# Supplementary material for: Diploid dual assemblies reveal the telocentric structure and extensive allelic heterogeneity of canine genomes
Source: NAR Genom Bioinform. 2026 Apr 23;8(2):lqag035. doi: 10.1093/nargab/lqag035 (PMC13103737; doi:10.1093/nargab/lqag035)
Supplement: lqag035_Supplemental_Files [file lqag035_supplemental_files.zip › SuppMaterial.pdf]

Supplementary Tables

	Clu-1	mCanLor1.2	UU_Cfam_GSD_1.0
Accession	GCA_034620435.1	GCA_905319855.2	GCA_011100685.1
Single Copy Gene (%)	98.38	98.66	98.44
Gap-free chromosomes	13	5	0

Table S1 Summary of three published canine genome assemblies
The fraction of intact single copy genes represented in each assembly obtained using compleasm with the carnivora_odb12 database of protein coding genes and the number of chromosomes depicted as single contigs is shown.

Table S2 Segmental duplications identified by BISER
Available online as a separate file.

Table S3 Position of centromeres in the ACD.1 assembly based on CENP-A ChIP-seq read depth
Available online as a separate file.

Assembly	Gap Excluded Identity	Gap Compressed Identity
ACD.1	0.9993	0.9988
ACD.2	0.9990	0.9985
CC.1	0.9987	0.9980
CC.2	0.9986	0.9979
GW.1	0.9984	0.9977
GW.2	0.9984	0.9977
NGSD.1	0.9984	0.9977
NGSD.2	0.9984	0.9977
SH.1	0.9985	0.9978
SH.2	0.9985	0.9978

Table S4 Sequence identity between each assembly and a region of CanFam4/ UU_Cfam_GSD_1.0
Sequence alignments were generated between each assembly and a 2 Mb region of UU_Cfam_GSD_1.0 that does not overlap segmental duplications (chr1:61500001-63500000) using minimap2 version 2.26. Gap excluded identity does not consider any insertions or deletions. Gap compressed identity considers consecutive gaps as one difference.

Table S5 LINE-1 transduction analysis
Available online as a separate file.

SINECs

Assembly	SNPs vs GW.1	Estimated Divergence (generations)	SINEs not in GW.1	Estimated insertion rate (1/n births)	SINEs in GW.1, absent from sample	Estimated insertion rate (1/n births)
NGSD.1	4213256	220048.2677	15183	14.5	13344	16.5
NGSD.2	4207702	219758.1956	15122	14.5	13306	16.5
SH.1	4173807	217987.941	14281	15.3	13344	16.3
SH.2	4178447	218230.277	14281	15.3	13236	16.5
CC.1	4197898	219246.1562	14784	14.8	13376	16.4
CC.2	4172340	217911.3231	14714	14.8	13376	16.3
CD.1	4220709	220437.5201	14236	15.5	13398	16.5
CD.2	4211961	219980.633	14295	15.4	13342	16.5
GW.2	2437135	127285.7227	7809	16.3	7883	16.1

LINE-1s

Assembly	SNPs vs GW.1	Estimated Divergence (generations)	LINEs not in GW.1	Estimated insertion rate (1/n births)	LINEs in GW.1, absent from sample	Estimated insertion rate (1/n births)
NGSD.1	4213256	220048.2677	2069	106.4	1674	131.5
NGSD.2	4207702	219758.1956	2095	104.9	1643	133.8
SH.1	4173807	217987.941	1850	117.8	1655	131.7
SH.2	4178447	218230.277	1854	117.7	1638	133.2
CC.1	4197898	219246.1562	1930	113.6	1650	132.9
CC.2	4172340	217911.3231	1916	113.7	1644	132.5
CD.1	4220709	220437.5201	1908	115.5	1664	132.5
CD.2	4211961	219980.633	1884	116.8	1686	130.5
GW.2	2437135	127285.7227	955	133.3	972	131

Table S6 SINEC and LINE-1 insertion rates estimated from genome assembly comparisons
The estimated rate of SINEC and LINE-1 insertions per generation is shown across assemblies. For each assembly, a rate was estimated based on the elements present in the indicated assembly and absent in GW.1, and for elements present in GW.1 and absent in the indicated assembly. Values are calibrated based on a SNP mutation rate of $4.5*10^{-9}$/bp/generation.

Supplementary Figures

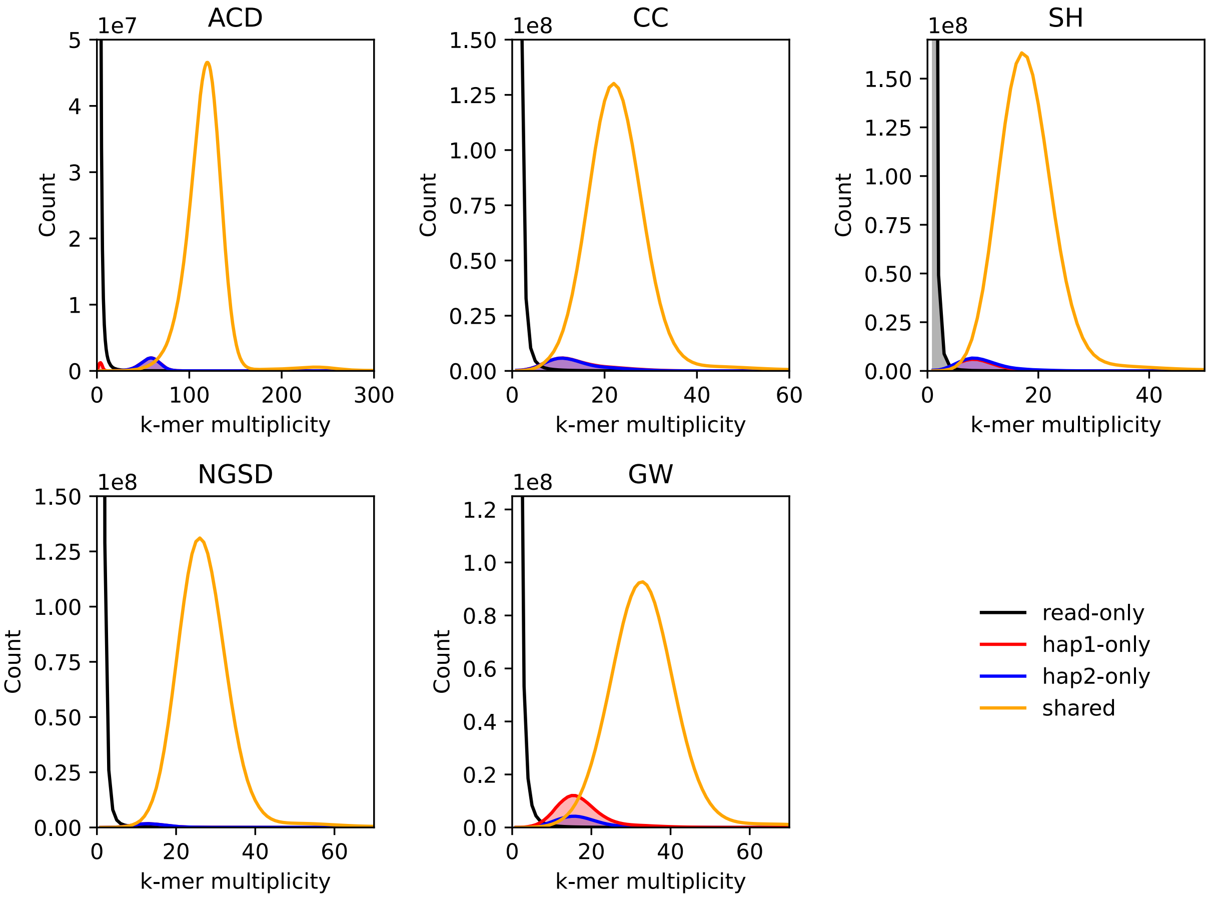

Figure S1 Assembly k-mer completeness spectrum plots

K-mer completeness profiles calculated using Merqury are shown for each of the five analyzed samples and their associated dual genome assemblies. K-mer presence was compared between the genome assemblies and the raw PacBio HiFi reads. In each block, the black histogram depicts k-mers found only in the reads, the red histogram depicts k-mers present only in the haplotype-1 assembly, the blue histogram depicts k-mers present only in the haplotype-2 assembly, and the orange histogram depicts k-mers present in both assemblies. GW is a male sample with the X chromosome represented in the haplotype-1 assembly.

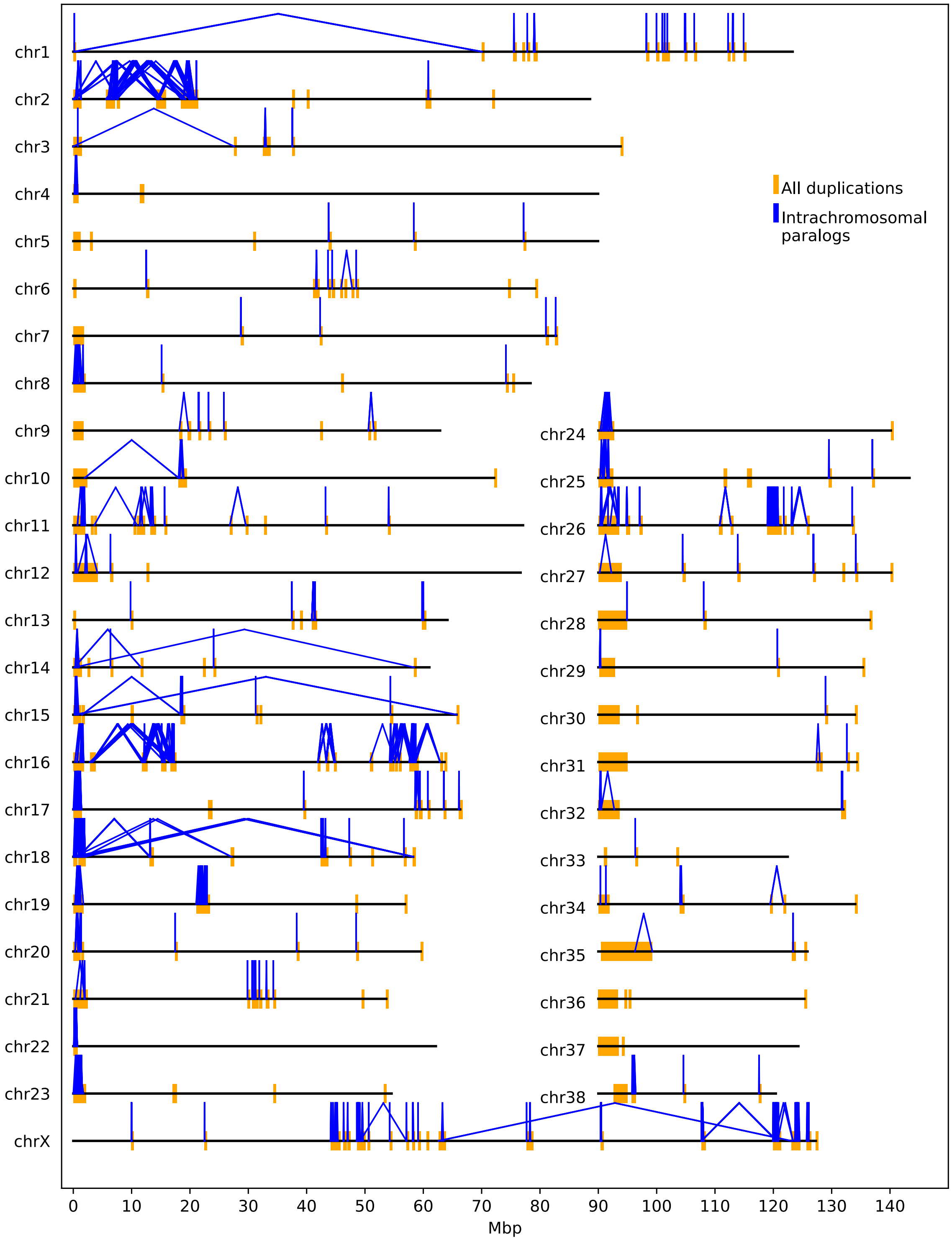

Figure S2 Landscape of segmental duplications in the ACD.1 assembly
Each chromosome is depicted as a horizontal black line. The position of segmental duplications at least 10 kb in size with at least 95% sequence identity is shown by gold rectangles. Blue lines connect intrachromosomal paralogs.

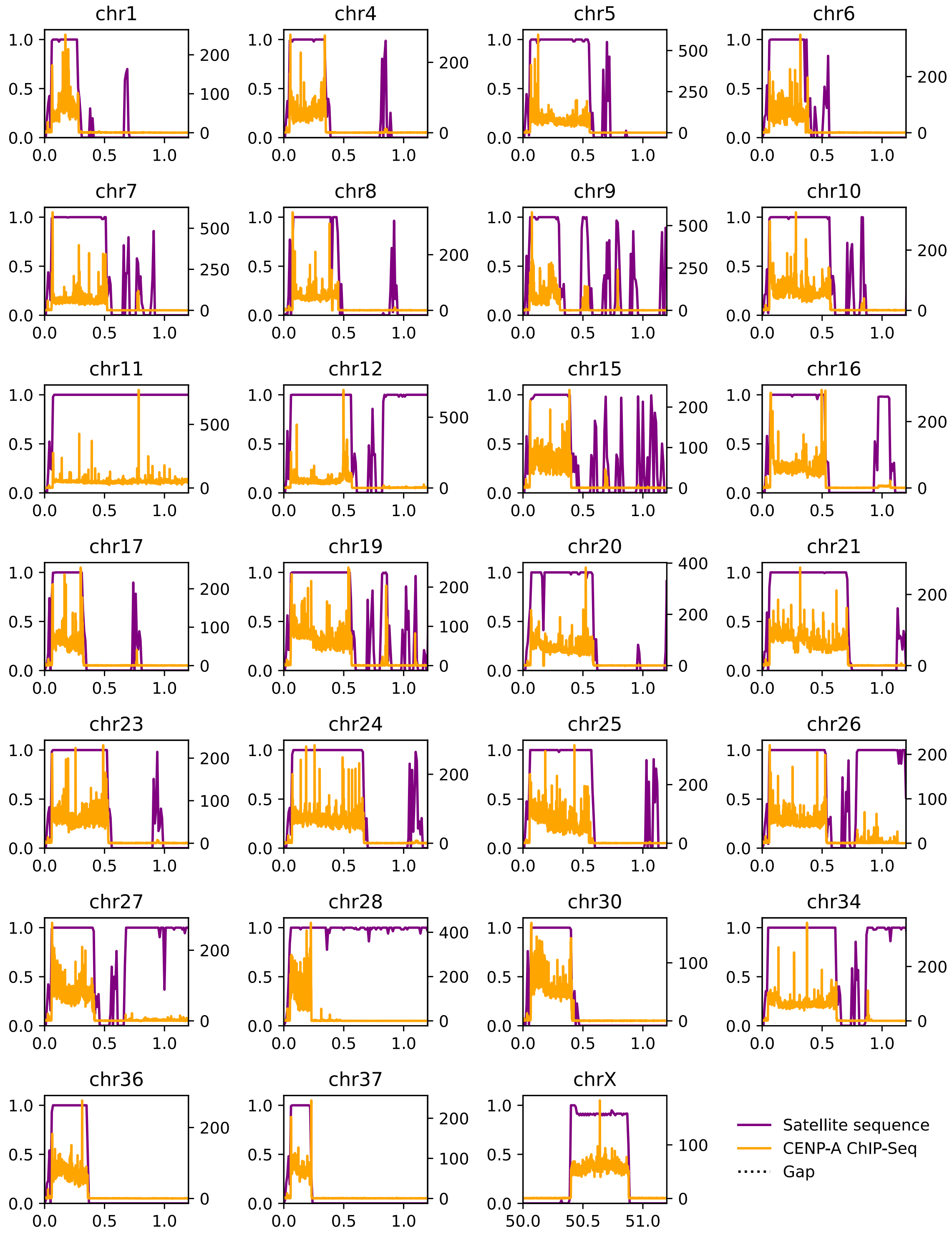

Figure S3 Overview of centromeres in the ACD.1 assembly
Features of the centromeres depicted for 27 assembled centromeres are shown. In each plot, the X-axis depicts chromosome position in Mbp. The purple line shows the fraction of sequence in 10 kb windows annotated as satellite sequence (left Y-axis scale) and the orange line depicts the average CENP-A Chip-Seq coverage in 1 kbp windows (right Y-axis scale).

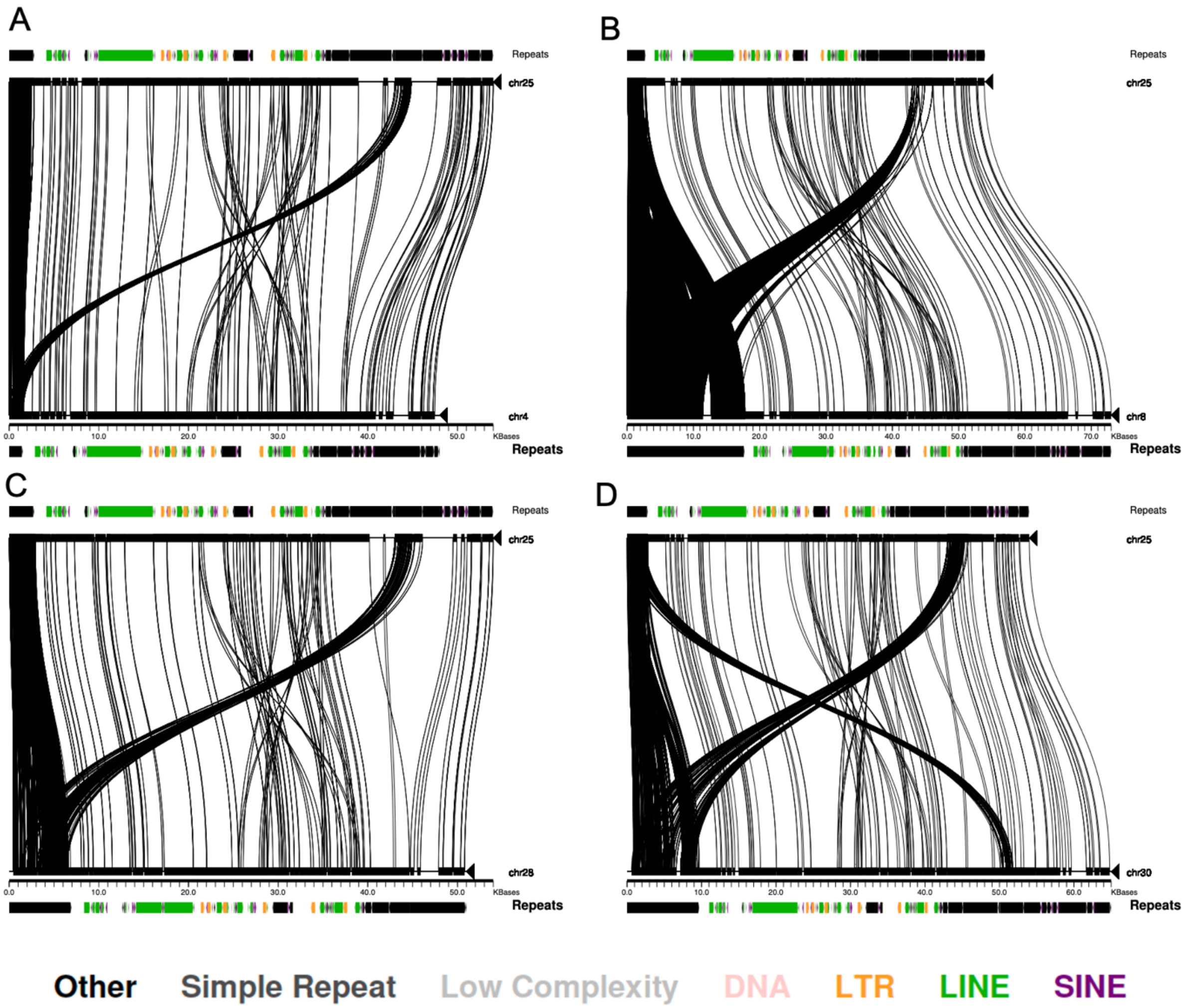

Figure S4 Comparison of telocentric chromosome ends from four canine chromosomes
Miropeats images are shown comparing the subtelomeric region of chr25 (position 1-54,000) versus (A) chr4:1-73,000, (B) chr8:1-73,000, (C) chr28:1-51,000 and (D) chr30:1-65,000. In each comparison, the matching sequence is connected by black boxes. The RepeatMasker annotation of each sequence is also plotted, with colors representing different repeat types as indicated. All sequences are from the ACD.1 assembly.

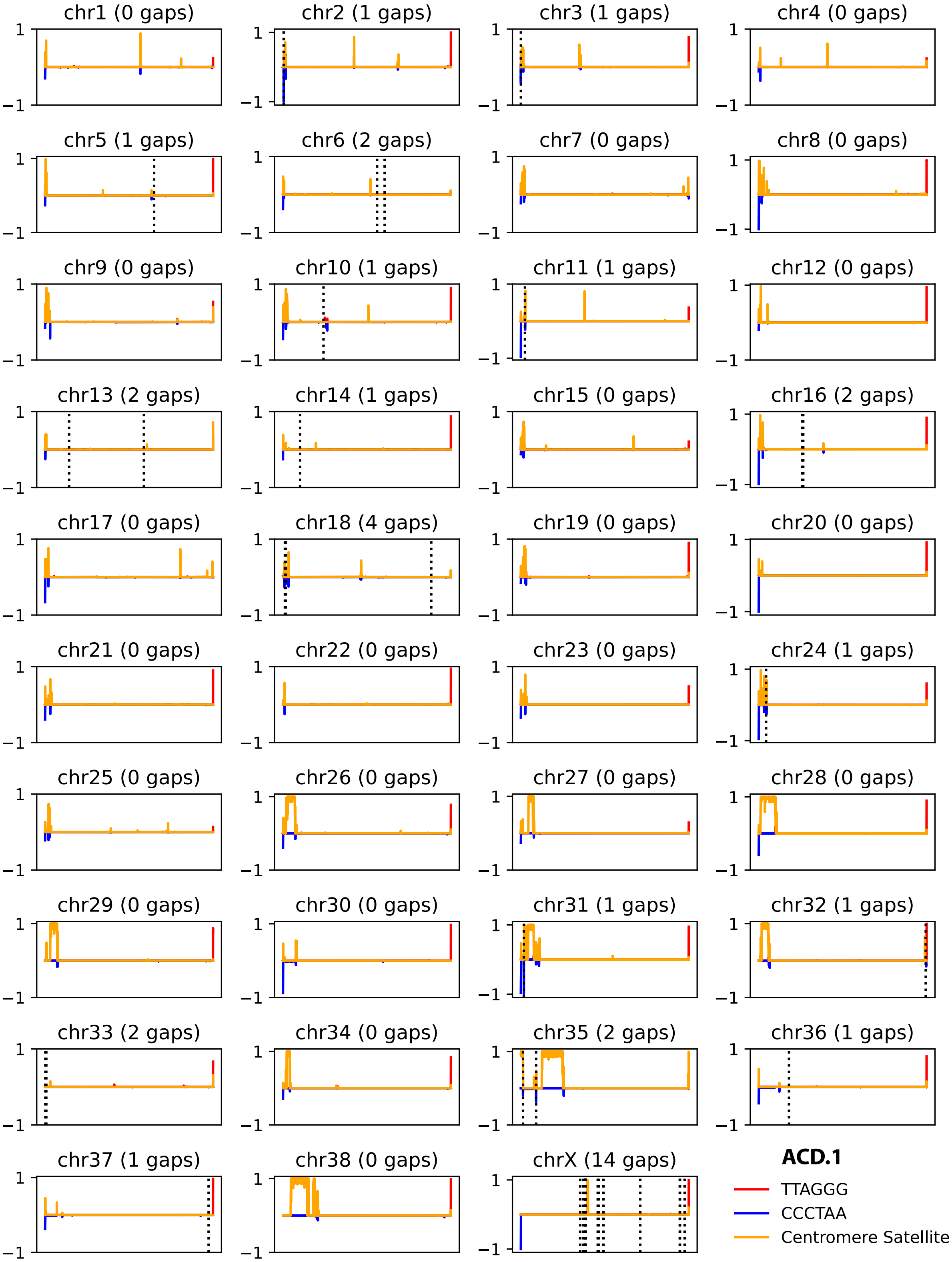
chr1 (0 gaps)
chr2 (1 gaps)
chr3 (1 gaps)
chr4 (0 gaps)
chr5 (1 gaps)
chr6 (2 gaps)
chr7 (0 gaps)
chr8 (0 gaps)
chr9 (0 gaps)
chr10 (1 gaps)
chr11 (1 gaps)
chr12 (0 gaps)
chr13 (2 gaps)
chr14 (1 gaps)
chr15 (0 gaps)
chr16 (2 gaps)
chr17 (0 gaps)
chr18 (4 gaps)
chr19 (0 gaps)
chr20 (0 gaps)
chr21 (0 gaps)
chr22 (0 gaps)
chr23 (0 gaps)
chr24 (1 gaps)
chr25 (0 gaps)
chr26 (0 gaps)
chr27 (0 gaps)
chr28 (0 gaps)
chr29 (0 gaps)
chr30 (0 gaps)
chr31 (1 gaps)
chr32 (1 gaps)
chr33 (2 gaps)
chr34 (0 gaps)
chr35 (2 gaps)
chr36 (1 gaps)
chr37 (1 gaps)
chr38 (0 gaps)
chrX (14 gaps)
ACD.1
TTAGGG
CCCTAA
Centromere Satellite

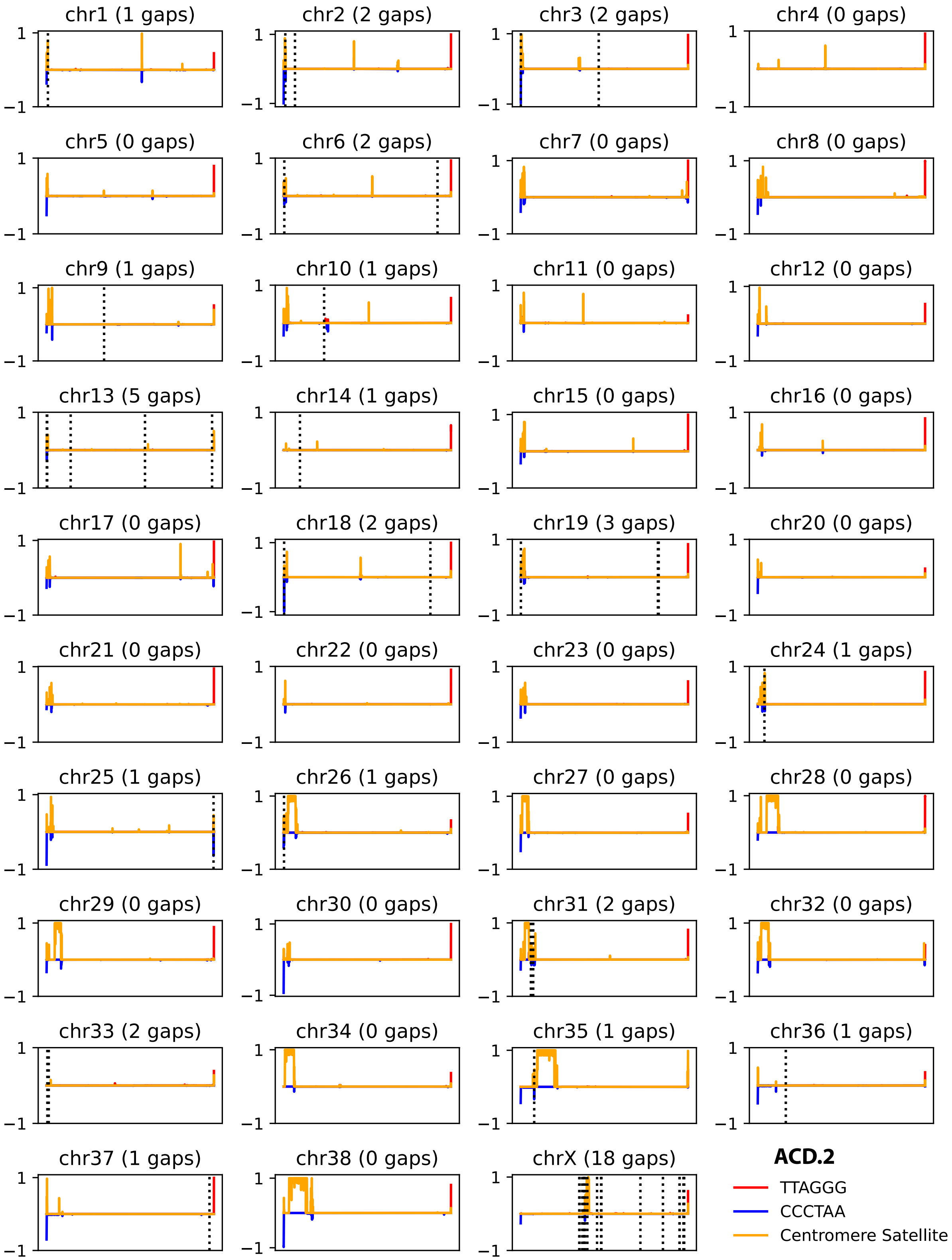
chr1 (1 gaps)
chr2 (2 gaps)
chr3 (2 gaps)
chr4 (0 gaps)
chr5 (0 gaps)
chr6 (2 gaps)
chr7 (0 gaps)
chr8 (0 gaps)
chr9 (1 gaps)
chr10 (1 gaps)
chr11 (0 gaps)
chr12 (0 gaps)
chr13 (5 gaps)
chr14 (1 gaps)
chr15 (0 gaps)
chr16 (0 gaps)
chr17 (0 gaps)
chr18 (2 gaps)
chr19 (3 gaps)
chr20 (0 gaps)
chr21 (0 gaps)
chr22 (0 gaps)
chr23 (0 gaps)
chr24 (1 gaps)
chr25 (1 gaps)
chr26 (1 gaps)
chr27 (0 gaps)
chr28 (0 gaps)
chr29 (0 gaps)
chr30 (0 gaps)
chr31 (2 gaps)
chr32 (0 gaps)
chr33 (2 gaps)
chr34 (0 gaps)
chr35 (1 gaps)
chr36 (1 gaps)
chr37 (1 gaps)
chr38 (0 gaps)
chrX (18 gaps)
ACD.2
TTAGGG
CCCTAA
Centromere Satellite

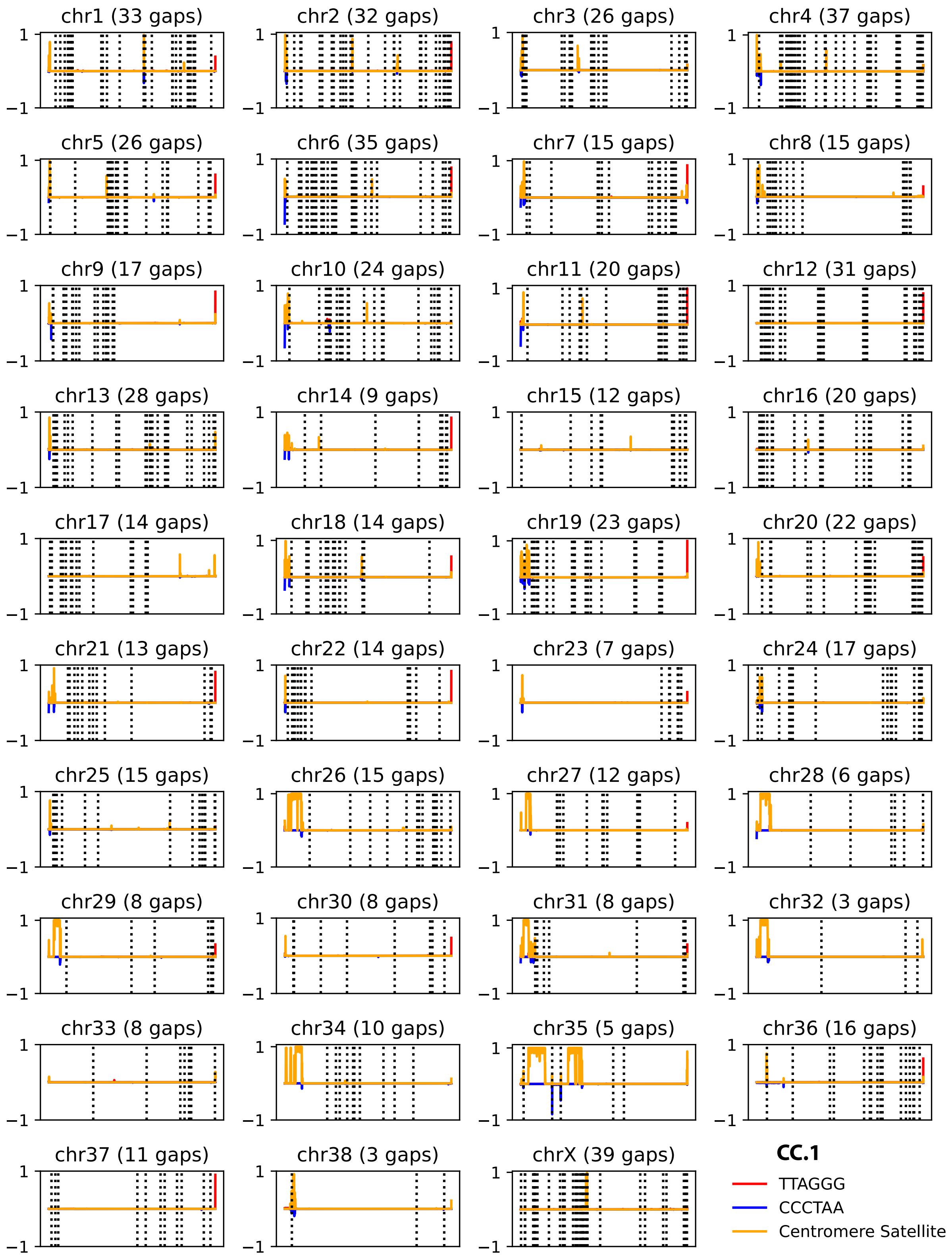

chr1 (33 gaps)
chr2 (32 gaps)
chr3 (26 gaps)
chr4 (37 gaps)
chr5 (26 gaps)
chr6 (35 gaps)
chr7 (15 gaps)
chr8 (15 gaps)
chr9 (17 gaps)
chr10 (24 gaps)
chr11 (20 gaps)
chr12 (31 gaps)
chr13 (28 gaps)
chr14 (9 gaps)
chr15 (12 gaps)
chr16 (20 gaps)
chr17 (14 gaps)
chr18 (14 gaps)
chr19 (23 gaps)
chr20 (22 gaps)
chr21 (13 gaps)
chr22 (14 gaps)
chr23 (7 gaps)
chr24 (17 gaps)
chr25 (15 gaps)
chr26 (15 gaps)
chr27 (12 gaps)
chr28 (6 gaps)
chr29 (8 gaps)
chr30 (8 gaps)
chr31 (8 gaps)
chr32 (3 gaps)
chr33 (8 gaps)
chr34 (10 gaps)
chr35 (5 gaps)
chr36 (16 gaps)
chr37 (11 gaps)
chr38 (3 gaps)
chrX (39 gaps)
CC.1
TTAGGG
CCCTAA
Centromere Satellite
1
−1

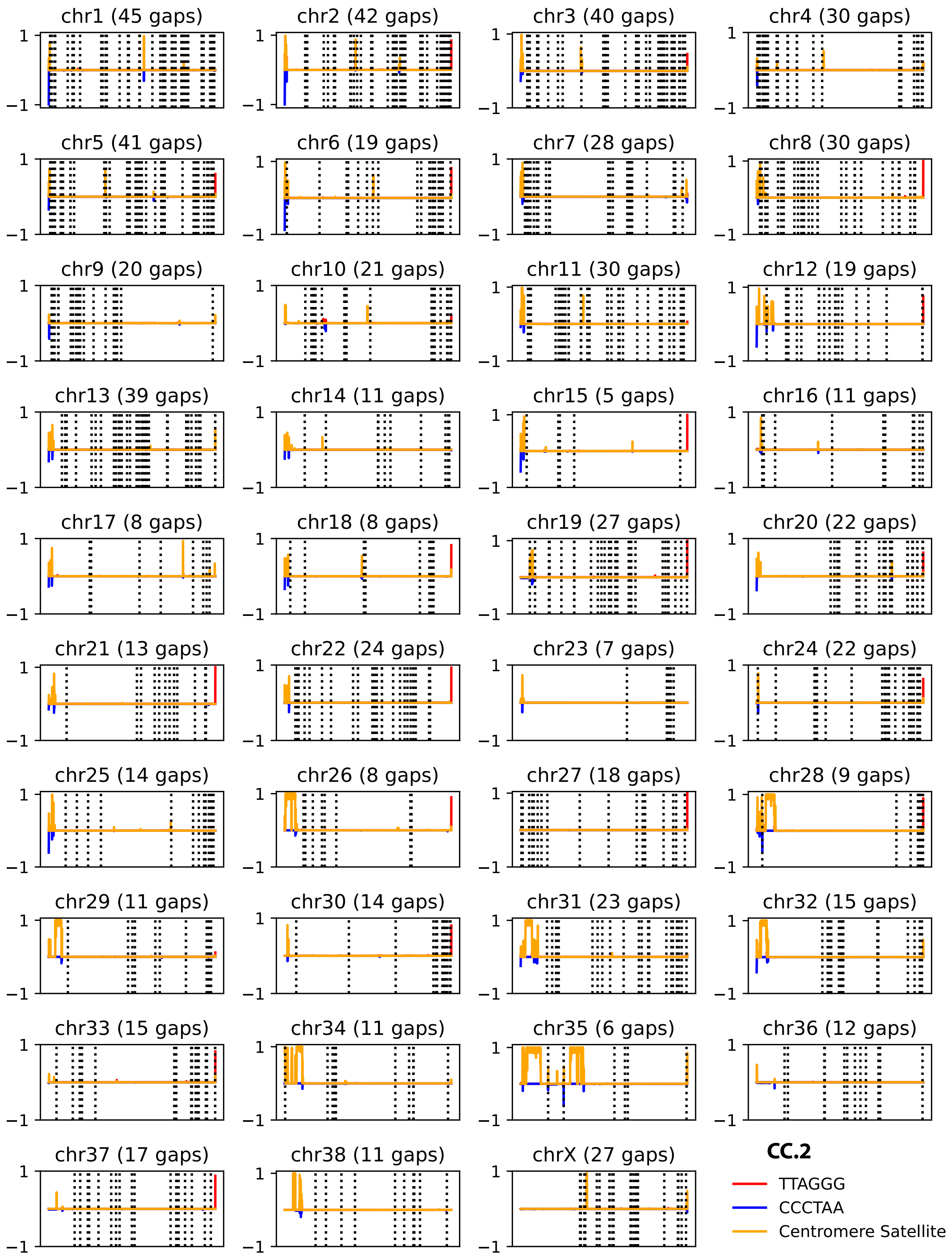
chr1 (45 gaps)
chr2 (42 gaps)
chr3 (40 gaps)
chr4 (30 gaps)
chr5 (41 gaps)
chr6 (19 gaps)
chr7 (28 gaps)
chr8 (30 gaps)
chr9 (20 gaps)
chr10 (21 gaps)
chr11 (30 gaps)
chr12 (19 gaps)
chr13 (39 gaps)
chr14 (11 gaps)
chr15 (5 gaps)
chr16 (11 gaps)
chr17 (8 gaps)
chr18 (8 gaps)
chr19 (27 gaps)
chr20 (22 gaps)
chr21 (13 gaps)
chr22 (24 gaps)
chr23 (7 gaps)
chr24 (22 gaps)
chr25 (14 gaps)
chr26 (8 gaps)
chr27 (18 gaps)
chr28 (9 gaps)
chr29 (11 gaps)
chr30 (14 gaps)
chr31 (23 gaps)
chr32 (15 gaps)
chr33 (15 gaps)
chr34 (11 gaps)
chr35 (6 gaps)
chr36 (12 gaps)
chr37 (17 gaps)
chr38 (11 gaps)
chrX (27 gaps)
CC.2
TTAGGG
CCCTAA
Centromere Satellite

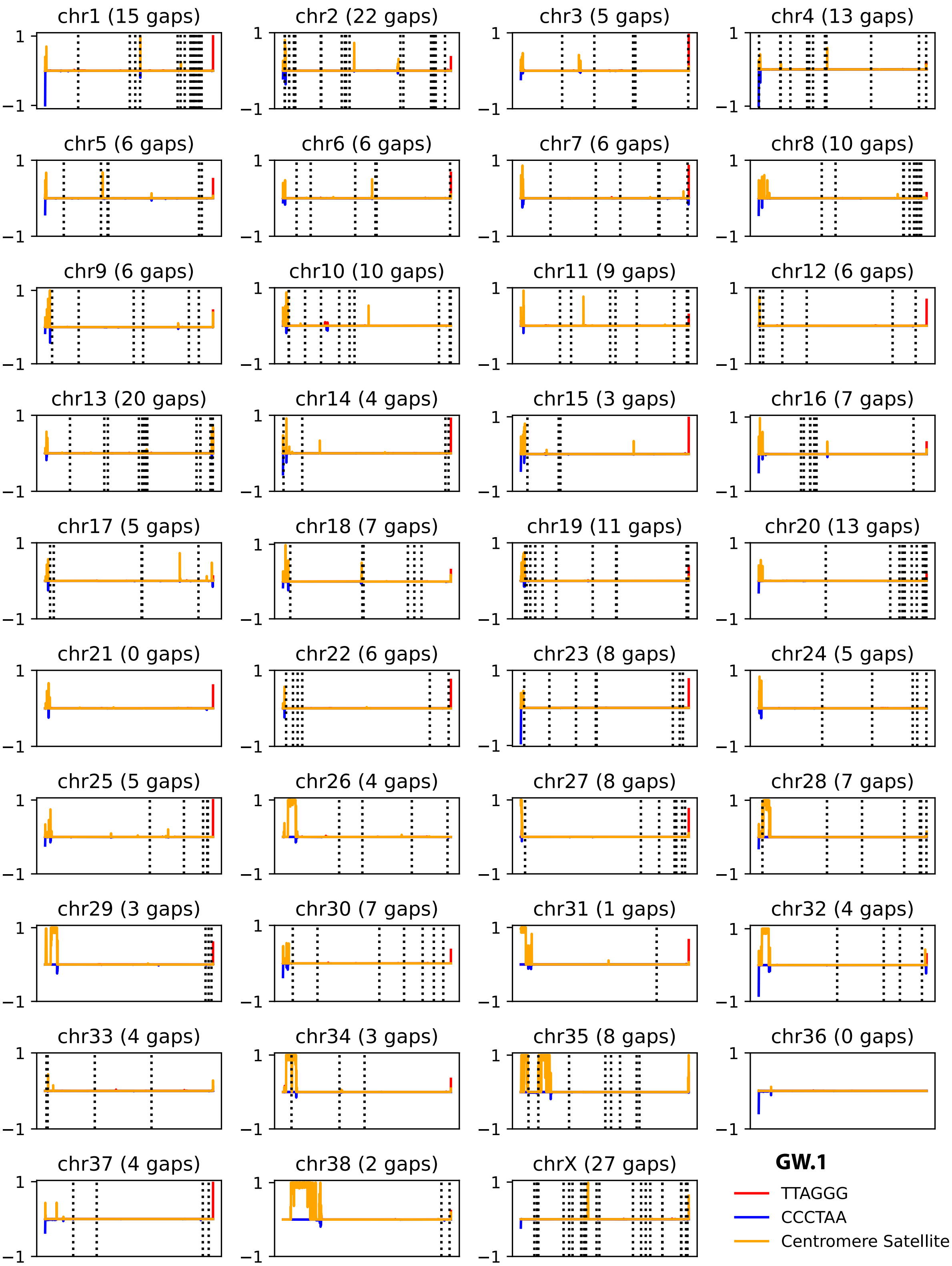

chr1 (15 gaps)
chr2 (22 gaps)
chr3 (5 gaps)
chr4 (13 gaps)
chr5 (6 gaps)
chr6 (6 gaps)
chr7 (6 gaps)
chr8 (10 gaps)
chr9 (6 gaps)
chr10 (10 gaps)
chr11 (9 gaps)
chr12 (6 gaps)
chr13 (20 gaps)
chr14 (4 gaps)
chr15 (3 gaps)
chr16 (7 gaps)
chr17 (5 gaps)
chr18 (7 gaps)
chr19 (11 gaps)
chr20 (13 gaps)
chr21 (0 gaps)
chr22 (6 gaps)
chr23 (8 gaps)
chr24 (5 gaps)
chr25 (5 gaps)
chr26 (4 gaps)
chr27 (8 gaps)
chr28 (7 gaps)
chr29 (3 gaps)
chr30 (7 gaps)
chr31 (1 gaps)
chr32 (4 gaps)
chr33 (4 gaps)
chr34 (3 gaps)
chr35 (8 gaps)
chr36 (0 gaps)
chr37 (4 gaps)
chr38 (2 gaps)
chrX (27 gaps)
1
−1
GW.1
TTAGGG
CCCTAA
Centromere Satellite

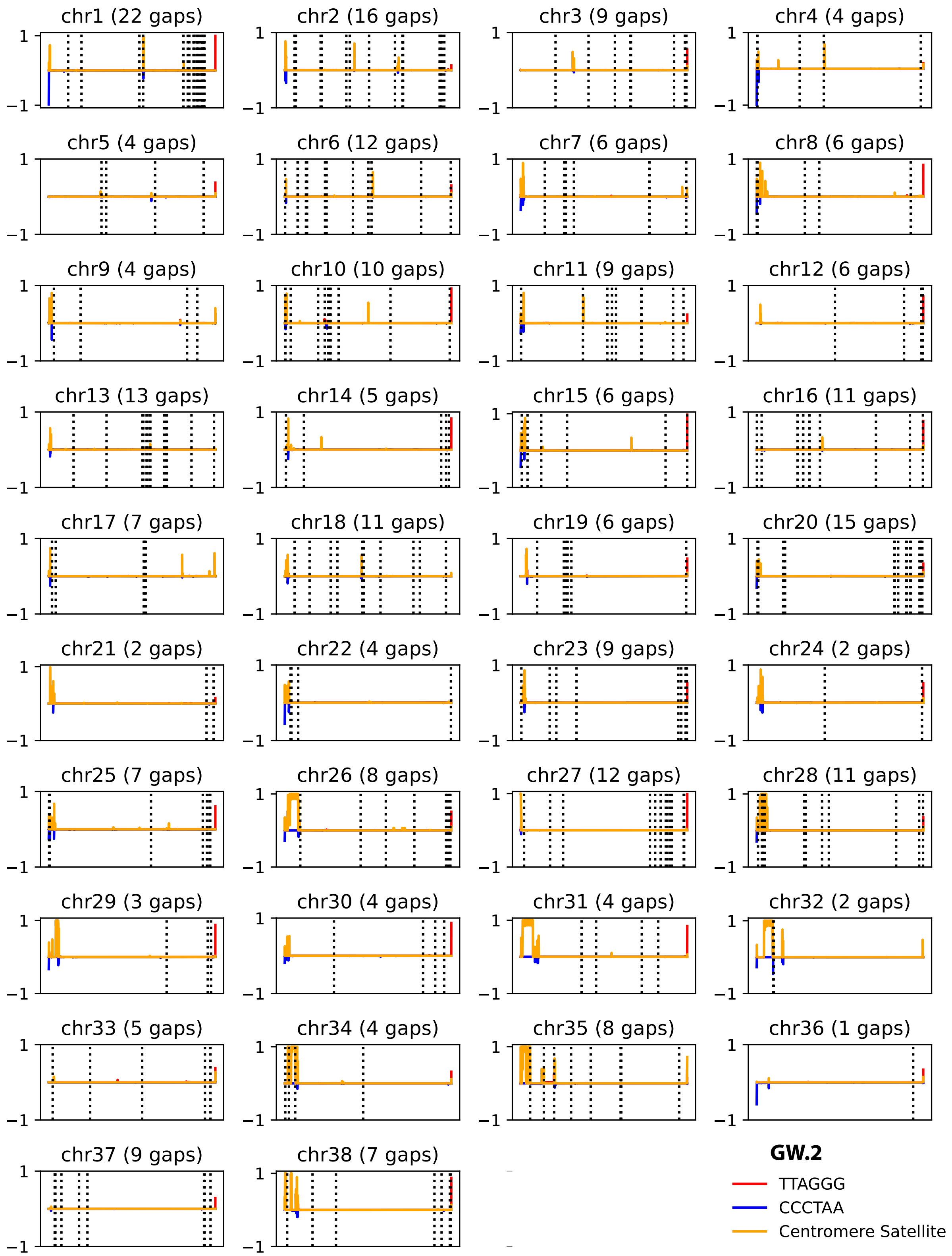
chr1 (22 gaps)
chr2 (16 gaps)
chr3 (9 gaps)
chr4 (4 gaps)
chr5 (4 gaps)
chr6 (12 gaps)
chr7 (6 gaps)
chr8 (6 gaps)
chr9 (4 gaps)
chr10 (10 gaps)
chr11 (9 gaps)
chr12 (6 gaps)
chr13 (13 gaps)
chr14 (5 gaps)
chr15 (6 gaps)
chr16 (11 gaps)
chr17 (7 gaps)
chr18 (11 gaps)
chr19 (6 gaps)
chr20 (15 gaps)
chr21 (2 gaps)
chr22 (4 gaps)
chr23 (9 gaps)
chr24 (2 gaps)
chr25 (7 gaps)
chr26 (8 gaps)
chr27 (12 gaps)
chr28 (11 gaps)
chr29 (3 gaps)
chr30 (4 gaps)
chr31 (4 gaps)
chr32 (2 gaps)
chr33 (5 gaps)
chr34 (4 gaps)
chr35 (8 gaps)
chr36 (1 gaps)
chr37 (9 gaps)
chr38 (7 gaps)
1
−1
GW.2
TTAGGG
CCCTAA
Centromere Satellite

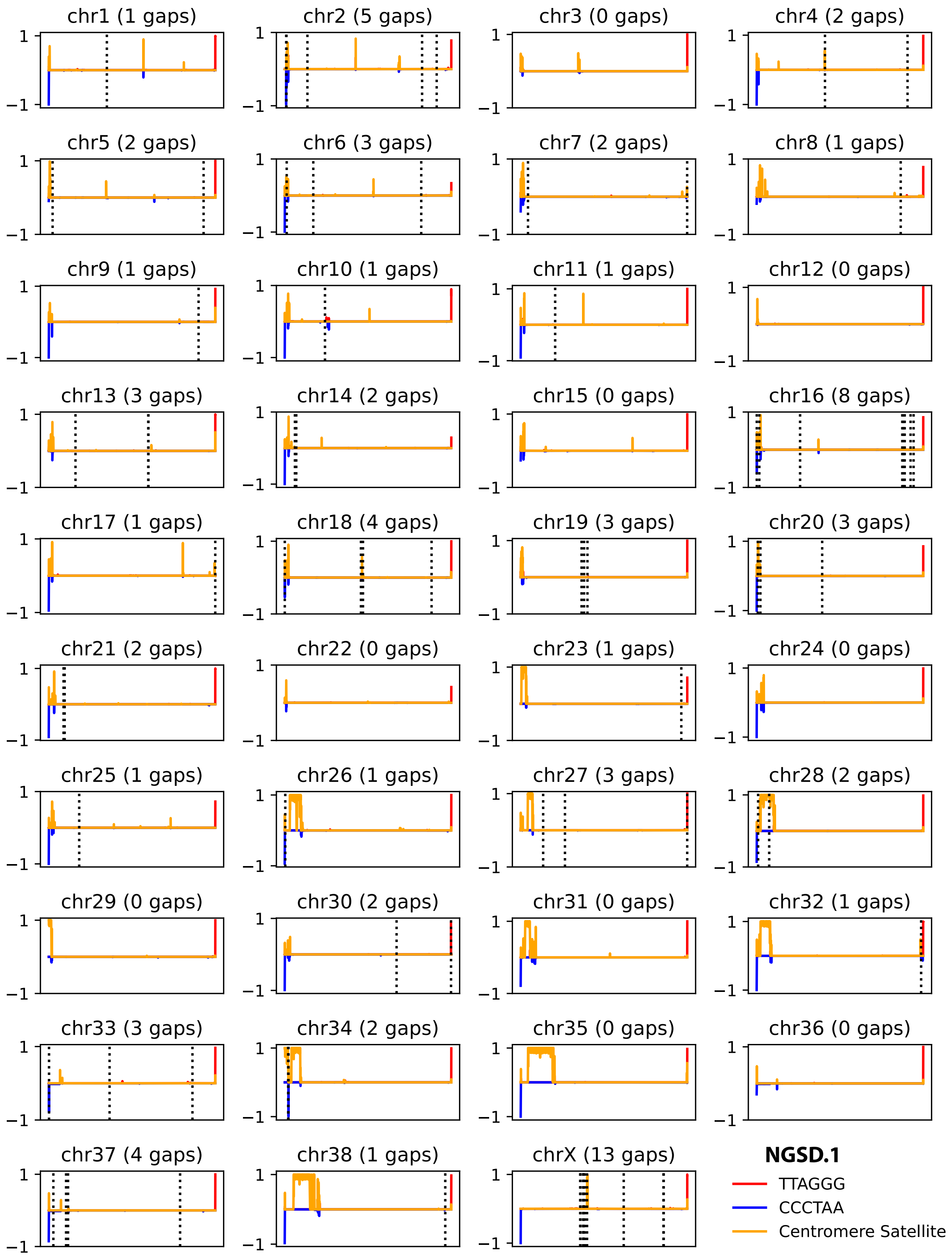
chr1 (1 gaps)
chr2 (5 gaps)
chr3 (0 gaps)
chr4 (2 gaps)
chr5 (2 gaps)
chr6 (3 gaps)
chr7 (2 gaps)
chr8 (1 gaps)
chr9 (1 gaps)
chr10 (1 gaps)
chr11 (1 gaps)
chr12 (0 gaps)
chr13 (3 gaps)
chr14 (2 gaps)
chr15 (0 gaps)
chr16 (8 gaps)
chr17 (1 gaps)
chr18 (4 gaps)
chr19 (3 gaps)
chr20 (3 gaps)
chr21 (2 gaps)
chr22 (0 gaps)
chr23 (1 gaps)
chr24 (0 gaps)
chr25 (1 gaps)
chr26 (1 gaps)
chr27 (3 gaps)
chr28 (2 gaps)
chr29 (0 gaps)
chr30 (2 gaps)
chr31 (0 gaps)
chr32 (1 gaps)
chr33 (3 gaps)
chr34 (2 gaps)
chr35 (0 gaps)
chr36 (0 gaps)
chr37 (4 gaps)
chr38 (1 gaps)
chrX (13 gaps)
NGSD.1
TTAGGG
CCCTAA
Centromere Satellite

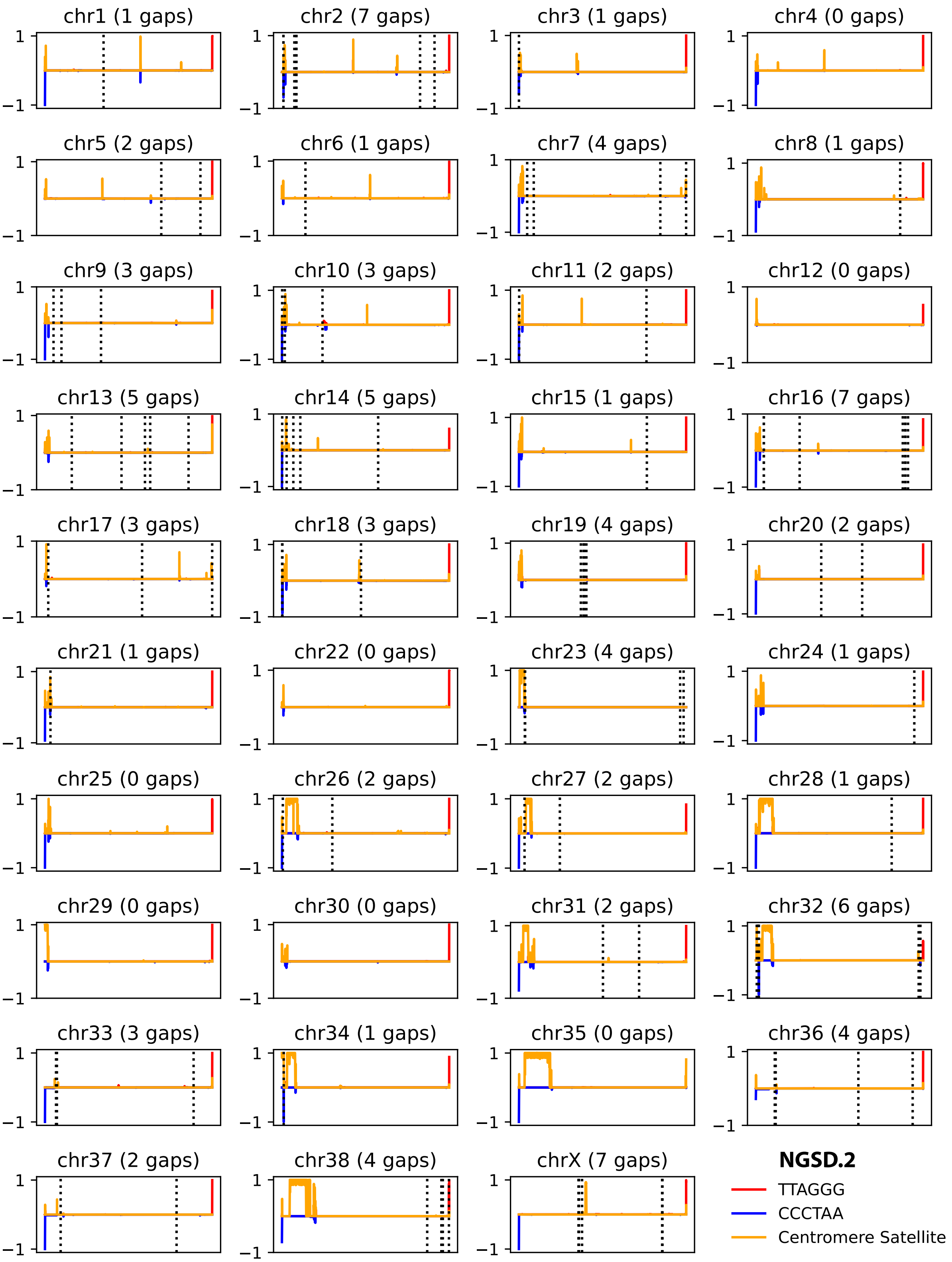
chr1 (1 gaps)
chr2 (7 gaps)
chr3 (1 gaps)
chr4 (0 gaps)
chr5 (2 gaps)
chr6 (1 gaps)
chr7 (4 gaps)
chr8 (1 gaps)
chr9 (3 gaps)
chr10 (3 gaps)
chr11 (2 gaps)
chr12 (0 gaps)
chr13 (5 gaps)
chr14 (5 gaps)
chr15 (1 gaps)
chr16 (7 gaps)
chr17 (3 gaps)
chr18 (3 gaps)
chr19 (4 gaps)
chr20 (2 gaps)
chr21 (1 gaps)
chr22 (0 gaps)
chr23 (4 gaps)
chr24 (1 gaps)
chr25 (0 gaps)
chr26 (2 gaps)
chr27 (2 gaps)
chr28 (1 gaps)
chr29 (0 gaps)
chr30 (0 gaps)
chr31 (2 gaps)
chr32 (6 gaps)
chr33 (3 gaps)
chr34 (1 gaps)
chr35 (0 gaps)
chr36 (4 gaps)
chr37 (2 gaps)
chr38 (4 gaps)
chrX (7 gaps)
NGSD.2
TTAGGG
CCCTAA
Centromere Satellite

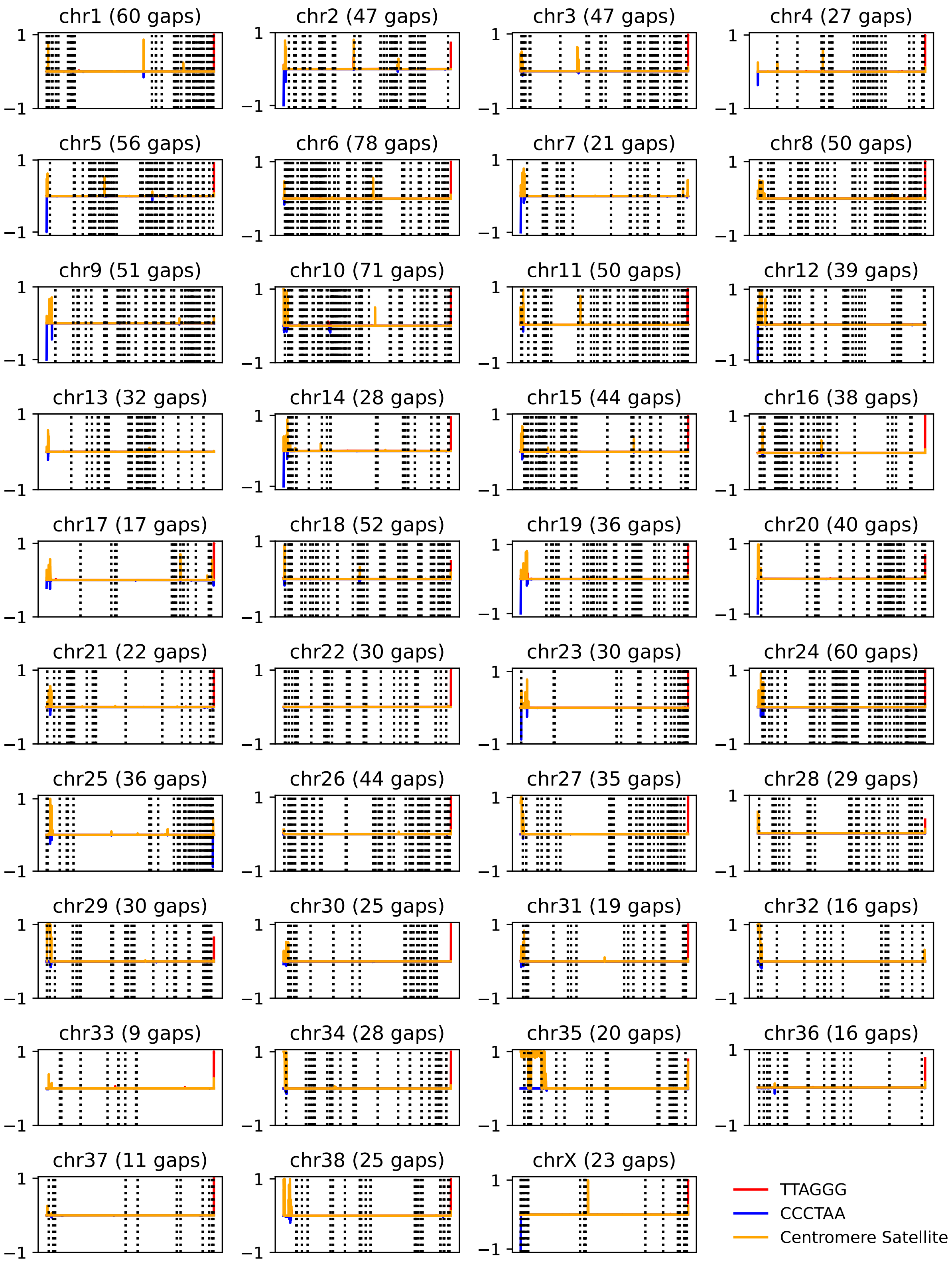
chr1 (60 gaps)
chr2 (47 gaps)
chr3 (47 gaps)
chr4 (27 gaps)
chr5 (56 gaps)
chr6 (78 gaps)
chr7 (21 gaps)
chr8 (50 gaps)
chr9 (51 gaps)
chr10 (71 gaps)
chr11 (50 gaps)
chr12 (39 gaps)
chr13 (32 gaps)
chr14 (28 gaps)
chr15 (44 gaps)
chr16 (38 gaps)
chr17 (17 gaps)
chr18 (52 gaps)
chr19 (36 gaps)
chr20 (40 gaps)
chr21 (22 gaps)
chr22 (30 gaps)
chr23 (30 gaps)
chr24 (60 gaps)
chr25 (36 gaps)
chr26 (44 gaps)
chr27 (35 gaps)
chr28 (29 gaps)
chr29 (30 gaps)
chr30 (25 gaps)
chr31 (19 gaps)
chr32 (16 gaps)
chr33 (9 gaps)
chr34 (28 gaps)
chr35 (20 gaps)
chr36 (16 gaps)
chr37 (11 gaps)
chr38 (25 gaps)
chrX (23 gaps)
1
−1
TTAGGG
CCCTAA
Centromere Satellite

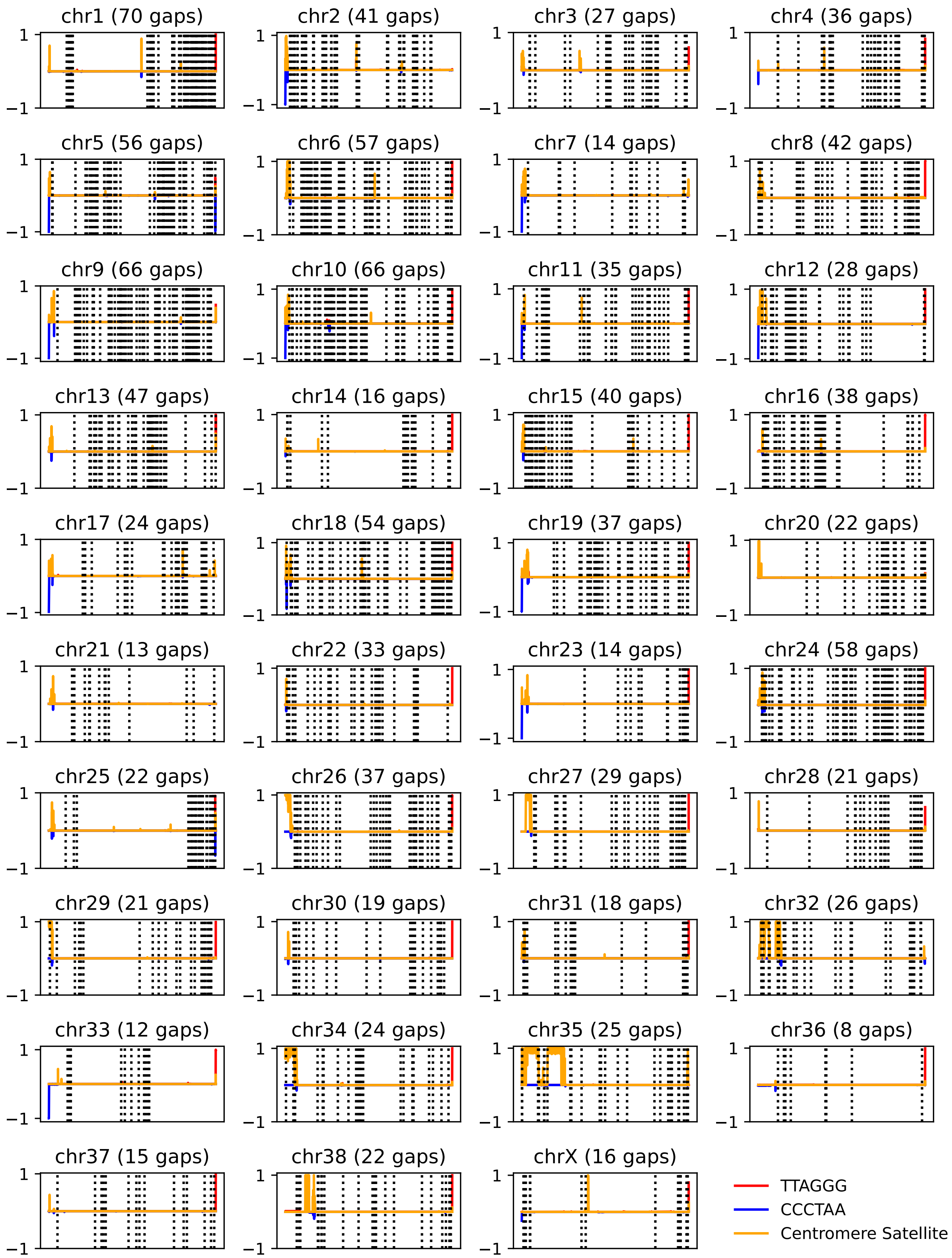
chr1 (70 gaps)
chr2 (41 gaps)
chr3 (27 gaps)
chr4 (36 gaps)
chr5 (56 gaps)
chr6 (57 gaps)
chr7 (14 gaps)
chr8 (42 gaps)
chr9 (66 gaps)
chr10 (66 gaps)
chr11 (35 gaps)
chr12 (28 gaps)
chr13 (47 gaps)
chr14 (16 gaps)
chr15 (40 gaps)
chr16 (38 gaps)
chr17 (24 gaps)
chr18 (54 gaps)
chr19 (37 gaps)
chr20 (22 gaps)
chr21 (13 gaps)
chr22 (33 gaps)
chr23 (14 gaps)
chr24 (58 gaps)
chr25 (22 gaps)
chr26 (37 gaps)
chr27 (29 gaps)
chr28 (21 gaps)
chr29 (21 gaps)
chr30 (19 gaps)
chr31 (18 gaps)
chr32 (26 gaps)
chr33 (12 gaps)
chr34 (24 gaps)
chr35 (25 gaps)
chr36 (8 gaps)
chr37 (15 gaps)
chr38 (22 gaps)
chrX (16 gaps)
1
−1
TTAGGG
CCCTAA
Centromere Satellite

Figure S5 Chromosomal overview of each assembly
A graphical depiction of chromosomal features is shown for each of the 10 assemblies. For each chromosome, the position of assembly gaps is shown with vertical dashed lines. The density of telomere repeats in each orientation is shown in red (positive values) and blue (negative values). The density of centromere satellites is plotted in yellow. All values are plotted in 10 kb windows along each chromosome. Note that since chromosomes differ in size the X-axis scale is not constant across plots.

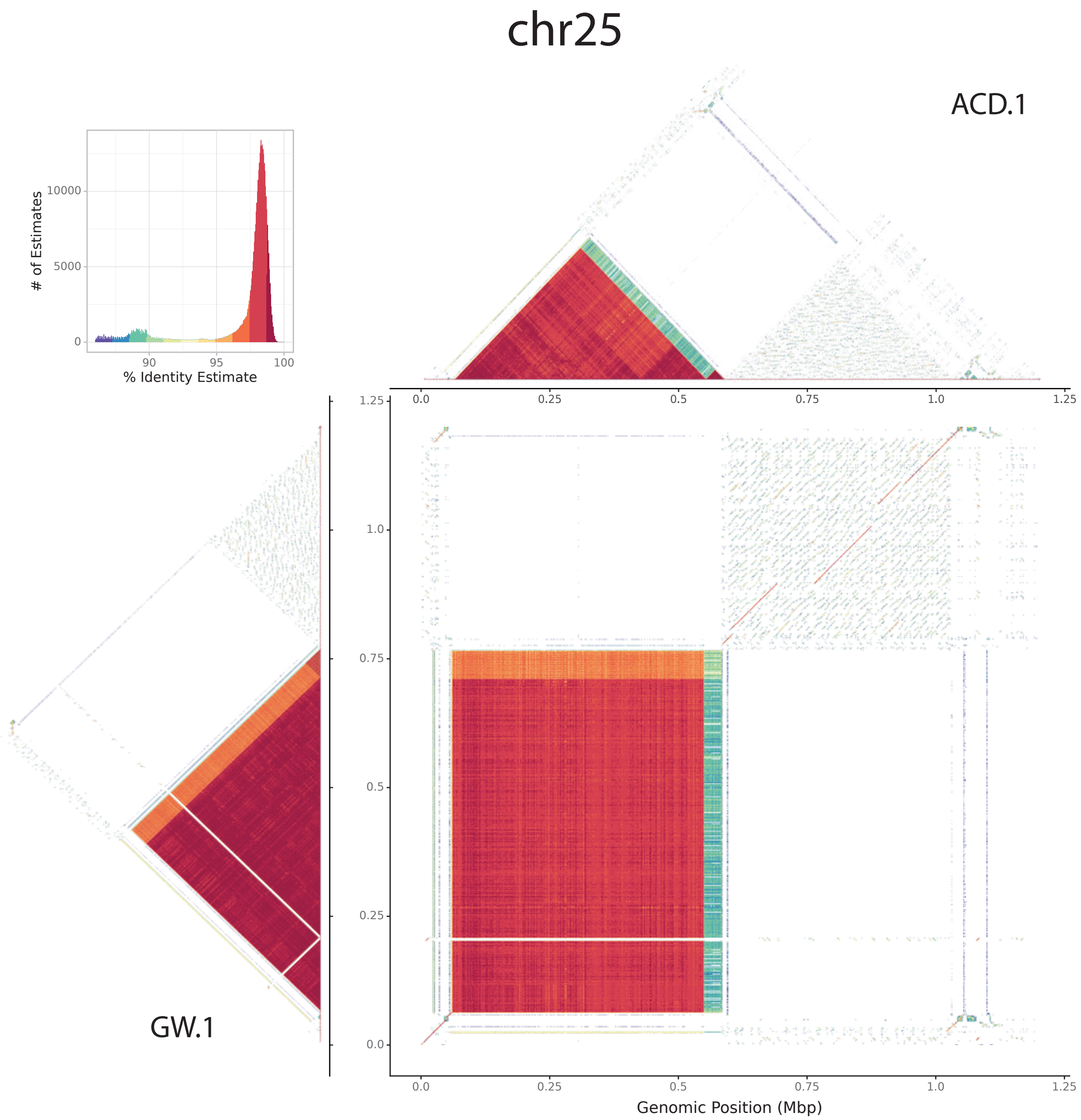

Figure S6 Comparison of chr25 centromere structure between ACD.1 and GW.1
A comparative dot plot of the first 1.2 Mb of chr25 from the ACD.1 (x-axis) and GW.1 (y-axis) assemblies, overlaid with self-identity plots, is shown. Plots were generated using ModDotPlot with a k-mer size of 21 and a window size of 1,000. This histogram insert shows the distribution of k-mer identities in the cross-assembly comparison. The break in the similarity heatmap in GW.1 corresponds to a 6.3 kb LINE-1 element present in GW.1 and absent in ACD.1 (coordinates: GW.1 chr25:203528-209838, family: L1MEc, divergence from consensus: 1.2%) that has a 16 bp target site duplication and a 34 bp poly(A) tail with one interruption.

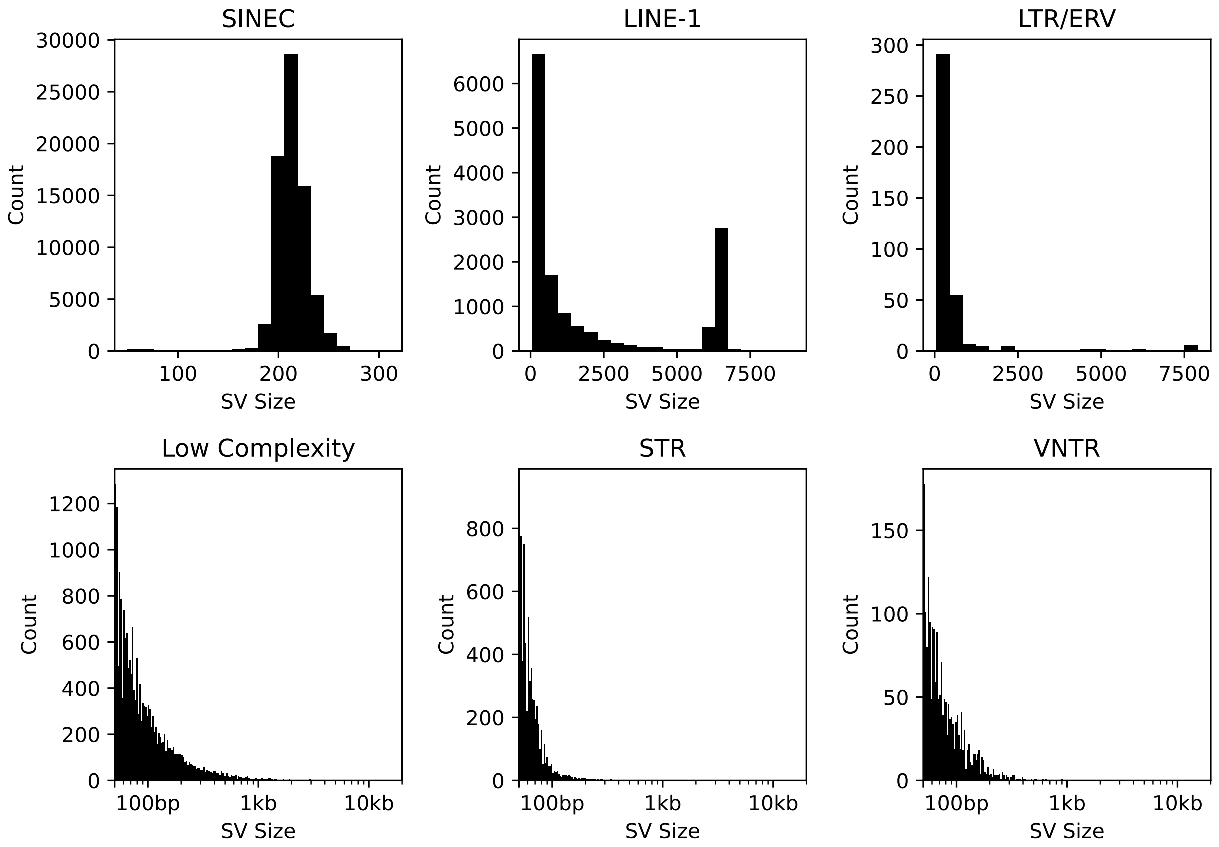

Figure S7 Structural variation size spectrum
Histograms of variant size are shown for structural variants classified into the SINEC, LINE-1, LTR/ERV, Low Complexity, STR, and VNTR categories. The top row consists of histograms of equally sized bins in linear space, while the bottom row consists of equally sized bins in log space.

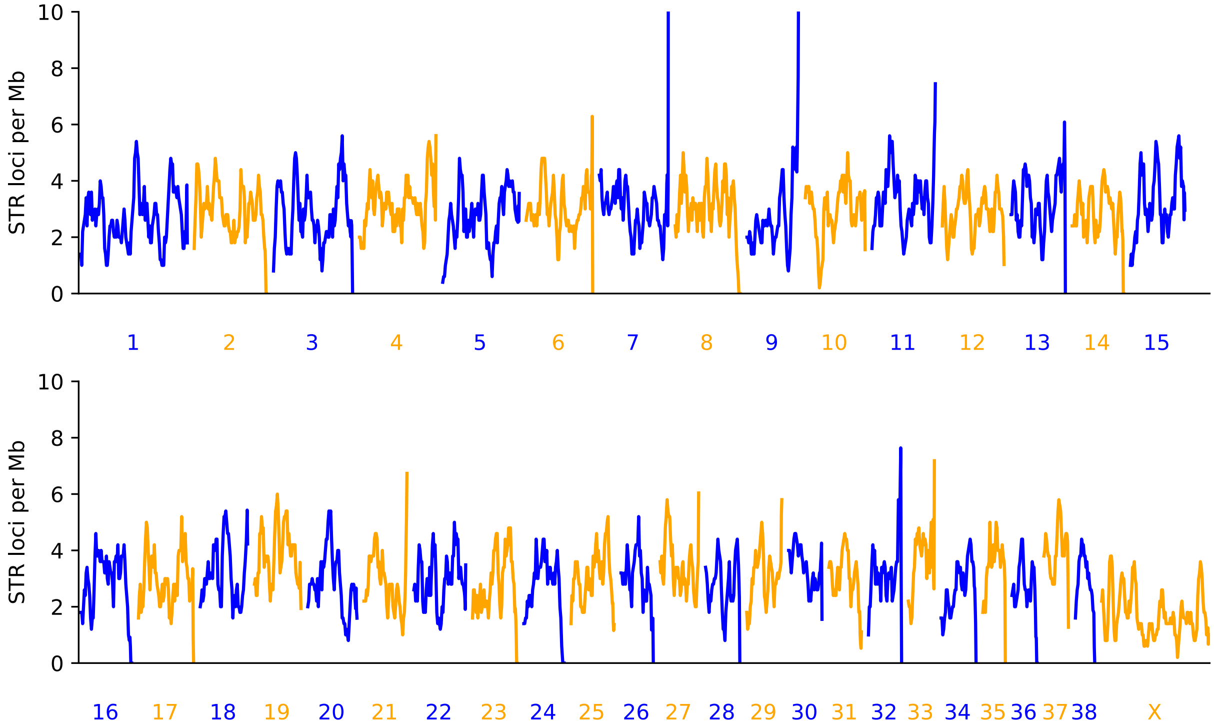

Figure S8 Landscape of STRs across the canine genome

The number of STR loci per Mb is shown across the canine genome. Values are plotted in sliding windows of size 5 Mb with a step size of 1 Mb across the 38 canine autosomes and the X chromosome. The X-axis scale is constant across plots. The Y axis is truncated to aid visualization; the final windows of chromosomes 7 and 9 have a STR density of 24.5 loci/Mb and 11.6 loci/Mb, respectively.

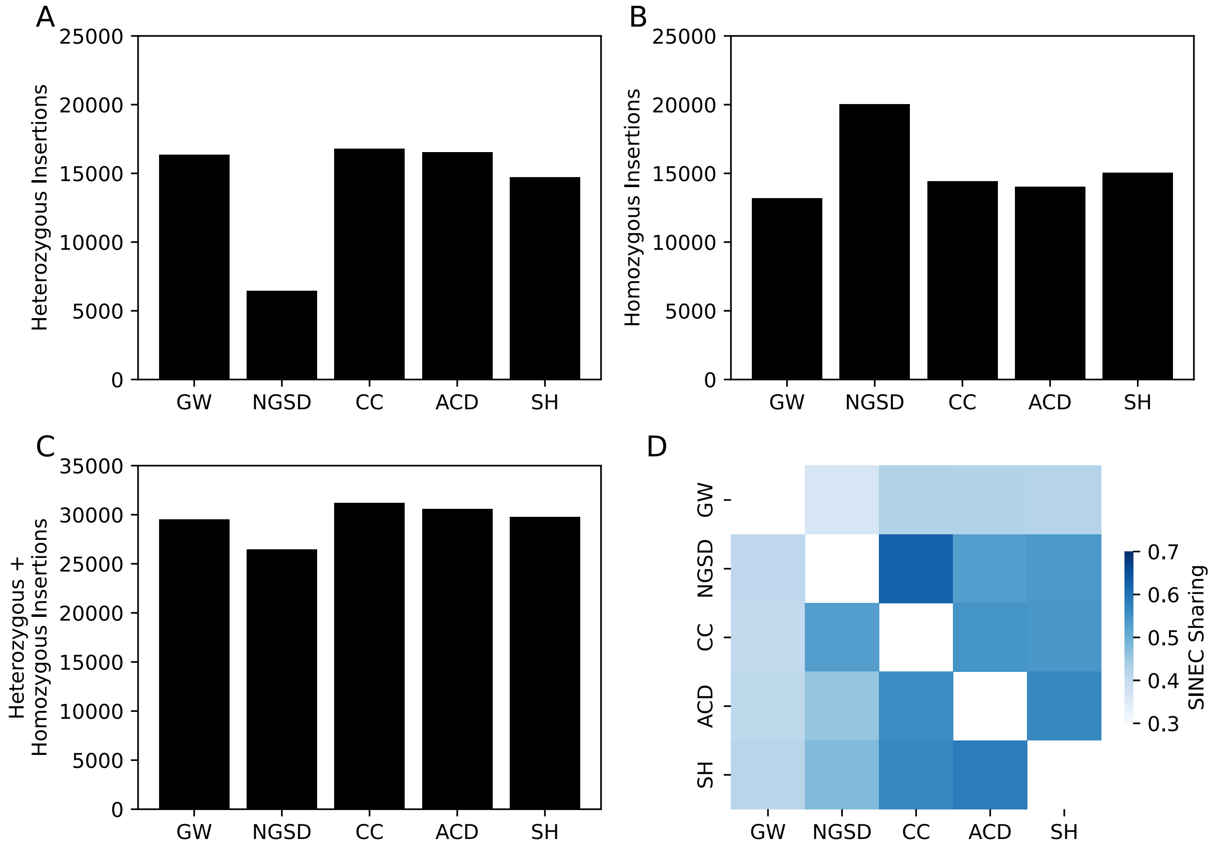

Figure S9 Sharing of autosomal dimorphic SINECs

We analyzed 69,384 autosomal SINEC loci that showed presence-absence dimorphism among the five analyzed samples. The number of loci where each sample is heterozygous (A) or homozygous (B) for the SINEC insertion is shown. The total count of heterozygous plus homozygous loci is shown in (C). The fraction of SINEC loci shared among sample is shown as a heatmap in (D). SINEC sharing was determined regardless of zygosity. Each square represents the fraction of SINECs present in the sample in row_i that are also found in the sample in $column_j$.

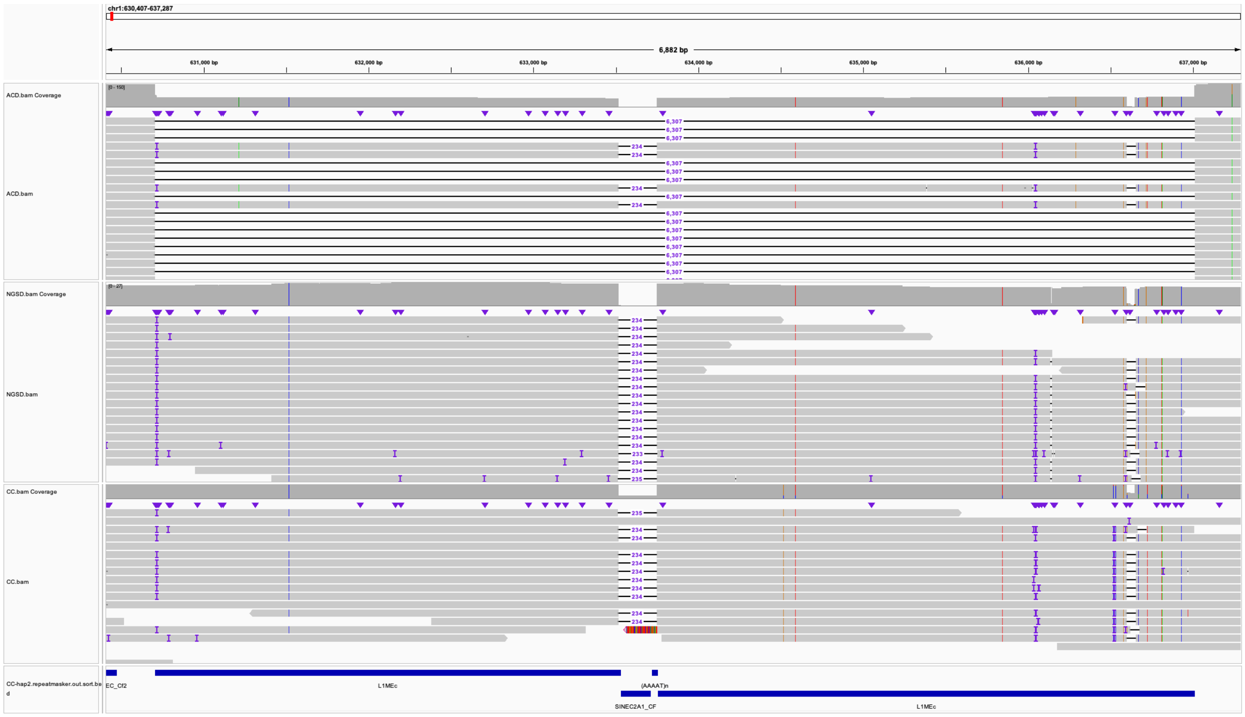

Figure S10 Aligned reads across the nested LINE-1/SINEC dimorphism support the assembled structures

An IGV view of aligned reads at the chr1:380121 locus is shown. PacBio HiFi reads were aligned to the CC.2 assembly. The bottom of the figure displays the RepeatMasker annotation of this sequence, which includes a LINE-1 element that also contains a SINEC sequence. HiFi reads from sample ACD (top) support the presence of the empty site allele as well as the LINE-1 without the SINEC insertion. Reads from NGSD support the presence of the LINE-1 without the SINEC insertion (middle). Reads from CC support the presence of the LINE-1 both with and without the SINEC insertion (bottom).
